# Structural features and phylogenetic implications of Cicadellidae subfamily and two new mitogenomes leafhoppers

**DOI:** 10.1371/journal.pone.0251207

**Published:** 2021-05-14

**Authors:** Xiaoxiao Chen, Zhouwei Yuan, Can Li, Christopher H. Dietrich, Yuehua Song

**Affiliations:** 1 School of Karst Science, Guizhou Normal University/State Key Laboratory Cultivation Base for Guizhou Karst Mountain Ecology Environment of China, Guiyang, China; 2 Guizhou Provincial Key Laboratory for Rare Animal and Economic Insect of the Mountainous Region, Guiyang University, Guiyang, China; 3 Illinois Natural History Survey, Prairie Research Institute, University of Illinois, Illinois, Champaign, United States of America; Sichuan University, CHINA

## Abstract

Complete mitochondrial genome sequences facilitate species identification and analyses of phylogenetic relationships. However, the available data are limited to the diverse and widespread insect family Cicadellidae. This study analyzes and summarizes the complete mitochondrial genome structure characteristics of 11 leafhopper subfamilies and two newly sequenced Typhlocybinae species, *Empoascanara wengangensis* and *E*. *gracilis*. Moreover, using 13PCGs and rRNA data to analyze the nucleotide diversity, evolution rate, and the phylogenetic relationship between the subfamilies of 56 species, verifying the taxonomic status analysis of *E*. *wengangensis* and *E*. *gracilis*. The analysis results show that the genome structures of the subfamilies and the newly sequenced two species are very similar, and the size of the CR region is significantly related to the repeat unit. However, in the entire AT-skews and CG-skews, the AT-skews of other subfamilies are all positive, and CG-skews are negative, while Empoascini of Typhlocybinae and Ledrinae are the opposite. Furthermore, among 13PCGs, the AT-skews of 13 species are all negative while CG-skews are positive, which from Empoascini in Typhlocybinae, Idiocerinae, Cicadellinae, Ledrinae, and Evacanthinae. Phylogenetic analysis shows that ML and PB analysis produce almost consistent topologies between different data sets and models, and some relationships are highly supported and remain unchanged. Mileewinae is a monophyletic group and is a sister group with Typhlocybinae, and the sister group of Evacanthinae is Ledrinae + Cicadellinae. Phylogenetic analysis grouped the two newly sequenced species with other species of Typhlocybinae, which was separated from other subfamilies, and all Erythroneurini insects gathered together. However, *E*. *gracilis* grouped into a single group, not grouped with species of the same genus (*Empoascanara*). This result does not match the traditional classification, and other nuclear genes or transcriptome genes may be needed to verify the result. Nucleotide diversity analysis shows that *nad4* and *nad5* may be evaluated as potential DNA markers defining the Cicadellidae insect species.

## Introduction

The insect mitochondrial genome (mtDNA) is the only extranuclear genetic information carrier in insects. It is usually a closed double-stranded DNA molecule with a measured molecular weight of about 15–20 kb. Usually, it contains 37 genes, including 13 protein coding genes (PCGs), NADH dehydrogenase 1–6 and 4L (*nad1-6* and *nad4L*), cytochrome c oxidase subunits 1–3 (*cox1-3*), ATPase subunit 6 and 8 (*atp6* and *atp8*), cytochrome b (*cytb*), two ribosomal RNAs genes (*16s* and *12s*) and 22 transfer RNA (tRNA) genes. A region rich in A + T, the control region, is also present [1, 2]. Due to the characteristics of simple structure, small molecular weight, stable composition, conservative arrangement, lack of recombination, maternal inheritance, relatively high evolutionary rate and easy detection, the mitochondrial genome has been widely used for species identification and population genetic research as well as in biogeography and phylogeny [3–5]. However, there are repetitive regions and AT-rich regions in mitochondrial genes, and the real mitochondrial sequence and nuclear copy mitochondrial sequence (pseudogene) have great similarities, making it difficult to assemble the mitochondrial gene correctly after sequencing. How to enrich mitochondrial DNA more effectively is worthy of our thinking. In addition, in the application of mitochondrial genes, whether it is a phylogenetic tree constructed or the study of population evolution based on the mitochondrial genome, a single protein coding gene and rRNA gene are commonly used. Compared with the complete mitochondrial genome, A single gene fragment can only reflect part of the effective biological information, and different researchers often get different results based on different genetic data, resulting in the phylogenetic relationship of many species of insects still unresolved. With the continuous development of sequencing technology, it is necessary to use the whole mitochondrial genome as much as possible for phylogenetic analysis., In order to get more accurate results.

The hemipteran insect family Cicadellidae (leafhoppers) includes >2,600 genera and >22,000 species worldwide, including >2,000 species in China [6, 7]. Erythroneurini, the larger tribe of the cicadellid subfamily Typhlocybinae, is widely distributed in the six major zoogeographic regions of the world and includes ~2,000 species worldwide and >300 species in China [8, 9]. All leafhoppers are phytophagous, different species feeding on a wide variety of plants, and the group includes critical agricultural pests and vectors of plant pathogens [10–12]. Simultaneously, because of its large number and small individuals, the taxonomic status and phylogenetic relationship between the subfamilies have been controversial, which has been discussed by related researchers. The study of the molecular phylogeny of Cicadellidae began in the 1990s. In 1993, Fang et al. sequenced and analyzed the *16S* of 19 genera 21 species of Deltocephalinae, and made a reasonable attempt to study the molecular phylogeny of leafhopper insects [13]. Subsequently, in 1995, Fang et al. combined molecular data and morphological characteristics, and based on *cytb* to conducted a branch analysis of the Deltocephalinae genera in the New North Territory, and the results still verified the monophyletic of the group [14]. Dietrich et al. conducted a systematic study on the phylogeny of Cicadaceae. Since 1997, phylogenetic studies have been conducted on Flexamia, Dalbulus, Membracoidea, *etc*., based on mitochondrial and nuclear gene fragments, and the relationship between leafhopper subfamilies, tribes, and genera has been analyzed. Most of the results are similar to those based on morphological characteristics [15–17]. Hereafter, Dietrich combined morphology and molecule, divided Cicadellidae into 27 subfamilies, and revised the Cicadellidae classification system proposed by Oman [5, 18]. However, these studies have not clearly established the relationship between the subfamilies in the Cicadellidae, and the relationship between some subfamilies and their relative groups remains to be explored [19, 20].

Although in recent years, the phylogenetic relationship of the entire Cicadellidae and a few subfamilies within it has been studied based on morphological or molecular biological information [21–24], but the overall understanding of leafhopper phylogeny is preliminary. These recent studies have partially revised the Cicadellidae classification of high-level elements, but more data are still needed to reconstruct and verify its phylogenetic relationship. Therefore, in this study, we newly sequenced and annotated two species to increase the molecular data of the Cicadellidae, and combined with the mitochondrial gene data of 13 protein-coding genes and two ribosomal RNA genes of 56 Cicadellidae insects from 11 subfamilies (Table 1) to reconstruct the phylogenetic relationship between these subfamilies, and confirm the taxonomic status of *Empoascanara wengangensis* (Chen & Song, 2020) and *E*. *gracilis* (Dworakowska, 1992) at the molecular level. Besides, we analyzed the mitochondrial structure of these two species and each subfamily, including genome size and nucleotide composition, codon usage, tRNA secondary structure, A + T control region repeat unit, nucleotide diversity and evolution rate, and compared the similarities and differences between the various subfamilies. It is hoped that this study can provide a reference for future research on leafhopper classification and phylogeny.

**Table 1:** List of the mitochondrial genomes analysed in the present study.

New mitochondrial genomes				
subFamily	Species	Length(bp)	GenBank No.	Reference
Typhlocybinae	*Empoascanara wengangensis*	14,830	MT445764	This study
	*Empoascanara gracilis*	14,627	MT576649	This study
	*Empoascanara dwalata*	15,271	MT350235.1	[25]
	*Empoascanara sipra*	14,827	MN604278.1	[26]
	*Mitjaevia protuberanta*	15,472	NC_047465.1	[27]
	*Limassolla lingchuanensis*	15,716	NC_046037.1	[28]
	*Paraahimia luodianensis*	16,497	NC_047464.1	[29]
	*Typhlocyba sp*.	15,223	KY039138.1	[30]
	*Parathailocyba orla*	15,382	MN894531.1	[31]
	*Zyginella minuta*	15,544	MT488436.1	[32]
	*Eupteryx minuscula*	16,944	MN910279.1	[33]
	*Bolanusoides shaanxiensis*	15,274	MN661136.1	Unpublished
	*Empoasca vitis*	15,154	NC_024838.1	[34]
	*Ghauriana sinensis*	15,491	MN699874.1	[35]
	*Empoasca flavescens*	15,152	MK211224.1	[36]
	*Empoasca onukii*	15,167	NC_037210.1	[37]
Deltocephalinae	*Pellucidus guizhouensis sp*.	16,555	MF784429.1	Unpublished
	*Phlogotettix sp*.	15,136	KY039135.1	[30]
	*Yanocephalus yanonis*	15,623	NC_036131.1	[30]
	*Scaphoideus maai*	15,188	KY817243.1	[38]
	*Scaphoideusi nigrivalveus*	15,235	KY817244.1	[38]
	*Scaphoideus varius*	15,207	KY817245.1	[38]
	*Tambocerus sp*.	15,955	KT827824.1	[39]
	*Maiestas dorsalis*	15,352	KX786285.1	[40]
	*Japananus hyalinus*	15,364	KY129954.1	[40]
	*Drabescoides nuchalis*	15,309	NC_028154.1	[41]
	*Macrosteles quadrimaculatus*	15,734	MG727894	[42]
	*Macrosteles quadrilineatus*	16,626	KY645960.1	[43]
	*Nephotettix cincticeps*	14,805	NC_026977.1	Unpublished
	*Paralaevicephalus gracilipenis*	16,114	MK450366.1	[44]
	*Watanabella graminea*	15,011	NC_045270.1	[45]
Idiocerinae	*Populicerus populi*	16,494	MH492318.1	[46]
	*Idioscopus myrica*	15,423	MH492317	[46]
	*Parocerus laurifoliae*	16,811	NC_039741.1	[46]
	*Idioscopus clypealis*	15,393	NC_039642.1	Unpublished
	*Idioscopus nitidulus*	15,287	NC_029203.1	Unpublished
Iassinae	*Batracomorphus lateprocessus*	15,356	NC_045858.1	[47]
	*Krisna concava*	14,304	NC_046067.1	[47]
	*Krisna rufimarginata*	14,724	NC_046068.1	[47]
	*Gessius rufidorsus*	14,634	MN577633.1	[47]
	*Trocnadella arisana*	15,131	NC_036480.1	[47]
	*Iassus dorsalis*	15,176	NC_046066.1	[47]
Cicadellinae	*Bothrogonia ferruginea*	15,262	KU167550.1	Unpublished
	*Homalodisca vitripennis*	15,304	AY875213.1	Unpublished
	*Cicadella viridis*	15,891	MK335936	[48]
Coelidiinae	*Taharana fasciana*	15,161	NC_036015.1	[49]
	*Olidiana ritcheriina*	15,166	NC_045207.1	[50]
Megophthalminae	*Japanagallia spinosa*	15,655	NC_035685.1	Unpublished
	*Durgades nigropicta*	15,974	NC_035684.1	Unpublished
Mileewinae	*Mileewa albovittata*	15,079	MK138358.1	[51]
Macropsinae	*Macropsis notata*	16,323	NC_042723.1	Unpublished
	*Oncopsis nigrofasciata*	15,927	MG813492.1	Unpublished
Ledrinae	*Ledra auditura*	16,094	MK387845.1	[52]
	*Tituria pyramidata*	15,331	NC_046701.1	Unpublished
Evacanthinae	*Evacanthus acuminatus*	14,793	MK948205.1	[53]
	*Evacanthus heimianus*	15,806	MG813486.1	[54]
Cercopoidea	*Callitettix braconoides*	15,637	NC_025497	[55]
(Family)	*Paphnutius ruficeps*	14,841	NC_021100	[56]
	*Cosmoscarta dorsimacula*	15,677	NC_040115	Unpublished

## Material and methods

### Sample collection and DNA extraction

Samples of *E*. *wengangensis* and *E*. *gracilis* were collected from Duyun (107°07′19′′-107°46′26′′ E, 25° 51′26′′-26°25′ 39′′ N) and Anshun (105°22′50′′-105°45′22′′ E, 25°33′38′′-25°55′32′′ N), Guizhou province, China, on 17 September 2018 and 13 May 2019. And leafhopper insects are not protected animals and are collected in non-natural reserves. The whole body was preserved in absolute ethanol and then stored at -20°C in the laboratory. After morphological identification, voucher specimens with male genitalia prepared were deposited in the insect specimen room of Guizhou Normal University. Total DNA was extracted from the entire body without the abdomen.

### Genome sequencing, assembly, and annotation

The mitochondrial gene sequence was obtained by sequencing. Primers were designed to amplify the mtDNA sequence in PCR reactions. The PCR reaction was performed using the LA Taq polymerase. The PCR conditions were as follows: initial denaturation 94°C for 2 min, then 35 cycles of denaturation at 94°C for 30 s, annealing at 55°C for 30 s, and extension at 72°C for 1 min/kb, followed by the final extension at 72°C for 10 min. The PCR products were sequenced directly, or if needed first cloned into a pMD18-T vector (Takara, JAP) and then sequenced, by the dideoxynucleotide procedure, ABI 3730 automatic sequencer (Sanger sequencing) using the same set of primers. After quality-proofing of the obtained fragments, the complete mt genome sequence was assembled manually using DNAStar [57], and the Blast function in NCBI performed homology search to verify the amplified sequence as the target sequence [58, 59]. The nucleotide base composition, codon usage and A + T content values were analyzed with MEGA 6.06 [60]. The secondary structure of tRNA genes was annotated using online tools tRNAscan-SE 1.21 [61] and ARWEN [62]. The tandem repeat sequence in the control area was determined by the online search tool Tandem Repeats Finder [63] and used Spss 22.0 software for correlation analysis. The base skew values for a given strand were calculated using the formulae [64]: AT-skew = [A-T]/[A+T] and GC-skew = [G-C]/[G+C]. The nucleotide diversity (Pi) and sliding window analysis (sliding window: 200 bp, step size: 20 bp) of 13 PCGs among 56 Cicadellidae species were conducted by DnaSP 5.0 software [65]. Furthermore, the ratio between the non-synonymous (Ka) and the synonymous substitution rate (Ks) of 13 PCGs was also estimated in DnaSP 5.0.

### Phylogenetic analysis

The phylogenetic analysis used the complete mitochondrial genomes of the two newly sequenced erythroneurine species plus 54 Cicadellidae species from our team and GenBank, and three outgroups of Cercopoidea (Table 1). The Gblocks Server online platform was used to eliminate poorly aligned positions and divergent regions of DNA protein alignment, and all alignments were checked and corrected in MEGA 6.06 [60] before phylogenetic analysis. Four datasets were generated: (1) 13 PCGs with 9262 nucleotides (PCGs); (2) the first and second codon positions of the 13 PCGs with 6174 nucleotides (PCG12); (3) 13 PCGs with 9262 nucleotides and two rRNA with 1219 nucleotides (PCGR); (4) and amino acid sequences of the 13 PCGs with 3289 amino acids (PCGAA).

The trimmed alignment was used to estimate the phylogeny by Bayesian inference (BI) using MrBayes 3.2.7 [66] and maximum likelihood (ML) using IQ-TREE [67]. BI selected GTR + I + G as the optimal model, running 10 million generations twice, sampling once every 1000 generations, after the average standard deviation of the segmentation frequency drops below 0.01, with the first 25% of the samples are discarded burn-in, and the remaining trees used to generate a consensus tree and calculate the posterior probability (PP) of each branch. ML constructed with the IQ-TREE used an ultrafast bootstrap approximation approach with 10,000 replicates and calculate bootstrap scores for each node (BP).

## Results and discussion

### Genome arrangement, organization and composition

In the Cicadellidae family, the structure and characteristics of the mitochondrial genomes of most leafhoppers are very similar, compared with the common gene rearrangements of Thysanoptera and Hymenoptera, the gene composition and arrangement of the mitochondrial genome of this family are relatively conservative. And the gene rearrangements are relatively rare, only three species have been reported from Deltocephalinae, and all rearrangements occur on the three genes trnW, trnC, and trnY in tRNA. Gene order of other species is the same as the putative ancestral insect (Drosophila yakuba) mitochondrial genome arrangement [1, 40, 42, 43, 68].

Among the 56 leafhoppers sequenced, the length of the mitochondrial genome ranges from 14 kbp to 17 kbp. The smallest is *Krisna concava* of Iassinae, while the largest is *Eupteryx minuscula* from Typhlocybinae, which is 14,304bp and 16,944bp in length, respectively. The average A+T content of the 11 subfamilies is 77.91% (A, 42.55%; T, 35.36%; C, 12.37%; G, 9.72%), of which Iassinae is the highest at 80.46%, while Ledrinae is the lowest at 75.94%. In all Cicadellidae species that have been sequenced, the content of base A > base T, base C > base G, except the six species from Ledrinae and Empoascini of Typhlocybinae, whose content is opposite. The range of AT-skews between subfamilies is -0.2246~0.1524, and the skew of GC-skews is -0.2179~0.1230. All species are positive for AT-skews and negative for GC-skews, except six species from Empoascini in Typhlocybinae and Ledrinae. Iassinae has the highest AT-skews, and Coelidiinae has the lowest GC-skews (Table 2 and S1 Table).

**Table 2:** Whole nucleotide compositions, AT- skews and GC-skews in 11 subfamilies of Cicadellidae.

Subfamily	A%	C%	G%	T%	A+T%	AT-skew	GC-skew
Typhlocybinae	42.20	12.15	9.88	35.77	77.97	0.0826	-0.1032
Empoascini (tribe)	**38.27**	**10.98**	**10.38**	**40.38**	**78.64**	**-0.0268**	**-0.0282**
Other tribes	43.52	12.54	9.71	34.23	77.75	0.1195	-0.1272
Deltocephalinae	42.45	13.40	9.75	34.39	76.84	0.1050	-0.1576
Idiocerinae	42.78	11.91	9.78	35.53	78.31	0.0925	-0.0983
Iassinae	46.36	11.20	8.34	34.10	80.46	0.1524	-0.1467
Cicadellinae	43.02	12.47	9.88	34.63	77.65	0.1081	-0.1156
Coelidiinae	44.89	13.44	8.63	33.04	77.93	0.1521	-0.2179
Megophthalminae	44.39	13.24	9.02	33.30	77.70	0.1427	-0.1897
Mileewinae	43.67	12.01	8.38	35.95	79.61	0.0970	-0.1783
Macropsinae	44.37	12.26	9.84	33.53	77.90	0.1392	-0.1096
Ledrinae	**29.44**	**10.55**	**13.51**	**46.50**	**75.94**	**-0.2246**	**0.1230**
Evacanthinae	40.39	10.92	9.73	38.96	79.35	0.0179	-0.0578

**Fig 1:**
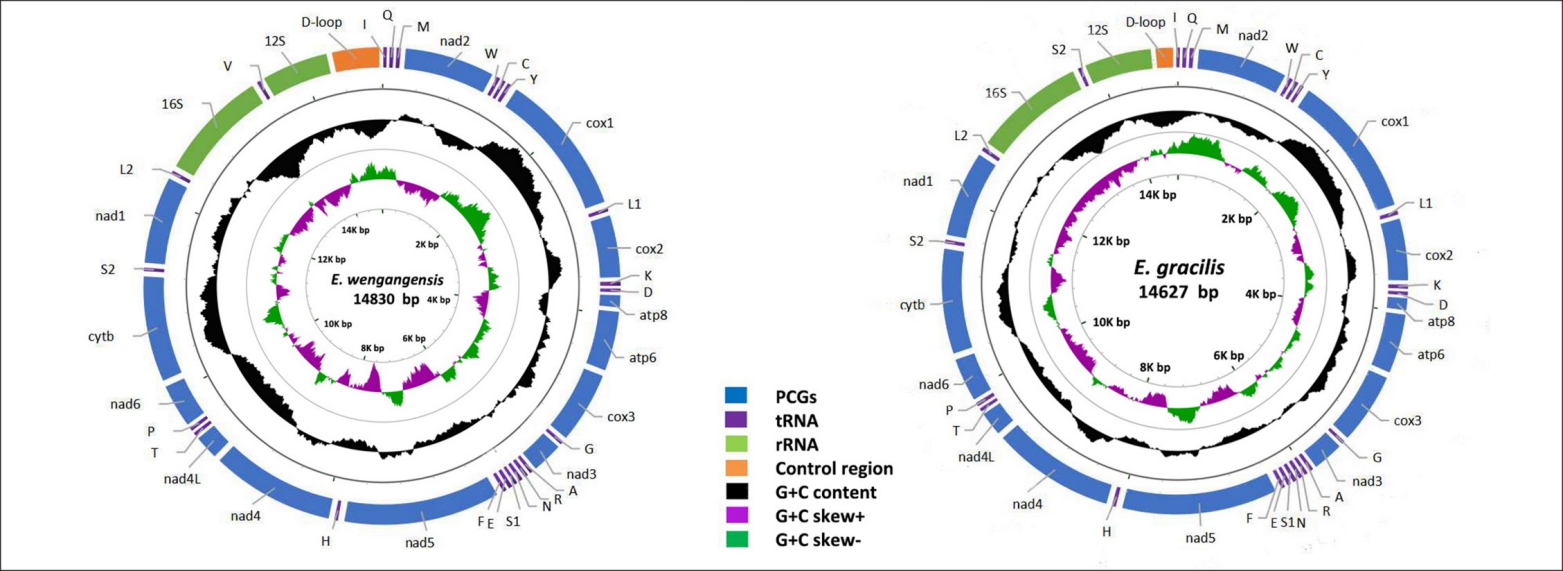
Circular map of the mitochondrial genome of *E*. *wengangensis* and *E*. *gracilis*.

Genome organization and nucleotide composition of the two new mitogenomes sequenced in this study are similar to those of other Erythroneurini reported previously [25–27]. The complete mitogenomes of *E*. *wengangensis* and *E*. *gracilis* are double-stranded plasmids with 14,830 and 14,627bp, respectively (Fig 1). Both contain the usual 13 PCGs, 22 tRNA genes, two rRNA genes, and a control region). Fourteen genes encode in the minority strand (L-strand) while the others encode in the majority strand (H-strand). *E*. *wengangensis* has a total of 45bp intergenic space in 12 regions ranging from 1 to 8bp. Eleven genes were found to overlap by a total of 47bp. *E*. *gracilis* has a total of 84bp intergenic space in 14 regions ranging from 2 to 15bp, and 11 genes were found to overlap by a total of 32bp (Table 3).

**Table 3:** Organization of the *E*. *wengangensis* and *E*. *gracilis* mitochondrial genome.

*E*. *wengangensis*/*E*. *gracilis*											
Gene	Position		Size(bp)		Intergenic		Start Codon		Stop Codon		Strand
tRNA-*Ile*	1–63	1–64	63	64	0	0					H
tRNA-*Gln*	61–129	62–131	69	70	-3						L
tRNA-*Met*	138–207	141–211	70	71	8	9					H
*nad2*	208–1179	212–1186	972	975	0	0	ATT		TAA		H
tRNA-*Trp*	1178–1242	1189–1254	65	66	-2	2					H
tRNA-*Cys*	1235–1296	1247–1311	62	65	-8	-8					L
tRNA-*Tyr*	1302–1368	1315–1377	67	63	5	3					L
*cox1*	1370–2905	1380–2924	1536	1545	1	2	ATG	ATT	TAA		H
tRNA-*Leu*	2906–2971	2927–2995	66	69	0	2					H
*cox2*	2972–3650	2996–3674	679		0	0	ATA		T		H
tRNA-*Lys*	3651–3721	3675–3745	71		0	0					H
tRNA-*Asp*	3721–3784	3745–3808	64		-1						H
*atp8*	3784–3936	3807–3959	153		-1	-2	TTG		TAA	TAG	H
*atp6*	3930–4580	3953–4606	651	654	-7		ATG		TAA		H
*cox3*	4583–5362	4615–5394	780		2	8	ATG		TAA	TAG	H
tRNA-*Gly*	5363–5424	5395–5456	62		0	0					H
*nad3*	5425–5778	5457–5810	354		0	0	ATA	ATT	TAA	TAG	H
tRNA-*Ala*	5783–5845	5809–5875	63	67	4	-2					H
tRNA-*Arg*	5848–5913	5891–5953	66	63	2	15					H
tRNA-*Asn*	5912–5976	5952–6018	65	67	-2						H
tRNA-*Ser*	5973–6032	6018–6085	60	68	-4	-1					H
tRNA-*Glu*	6041–6107	6097–6162	67	66	8	11					H
tRNA-*Phe*	6112–6177	6165–6231	66	67	4	2					L
*nad5*	6178–7849	6232–7903	1672		0	0	TTG		T		L
tRNA-*His*	7850–7912	7904–7972	63	69	0	0					L
*nad4*	7912–9237	7972–9297	1326		-7	-1	ATG		TAA		L
*nad4L*	9231–9509	9291–9569	279		1	-7	ATG		TAA	TAG	L
tRNA-*Thr*	9512–9575	9572–9633	64	62	2						H
tRNA-*Pro*	9576–9639	9641–9708	64	68	0	7					L
*nad6*	9642–10124	9714–10199	483	486	2	5	ATT	ATG	TAA		H
*cytb*	10132–11268	10200–11336	1137		7		ATG		TAG	TAA	H
tRNA-*Ser*	11267–11329	11346–11412	63	67	-2	9					H
*nad1*	11320–12261	11411–12346	942	936	-1	-2	ATT		TAA		L
tRNA-*Leu*	12262–12326	12347–12413	65	67	0	0					L
*16S*	12327–13514	12414–13615	1188	1202	0	0					L
tRNA-*Val*	13515–13578	13616–13680	64	65	0	0					L
*12S*	13579–14305	13681–14415	727	735	0	0					L
D-loop	14306–14830	14416–14627	525	212							

**Fig 2:**
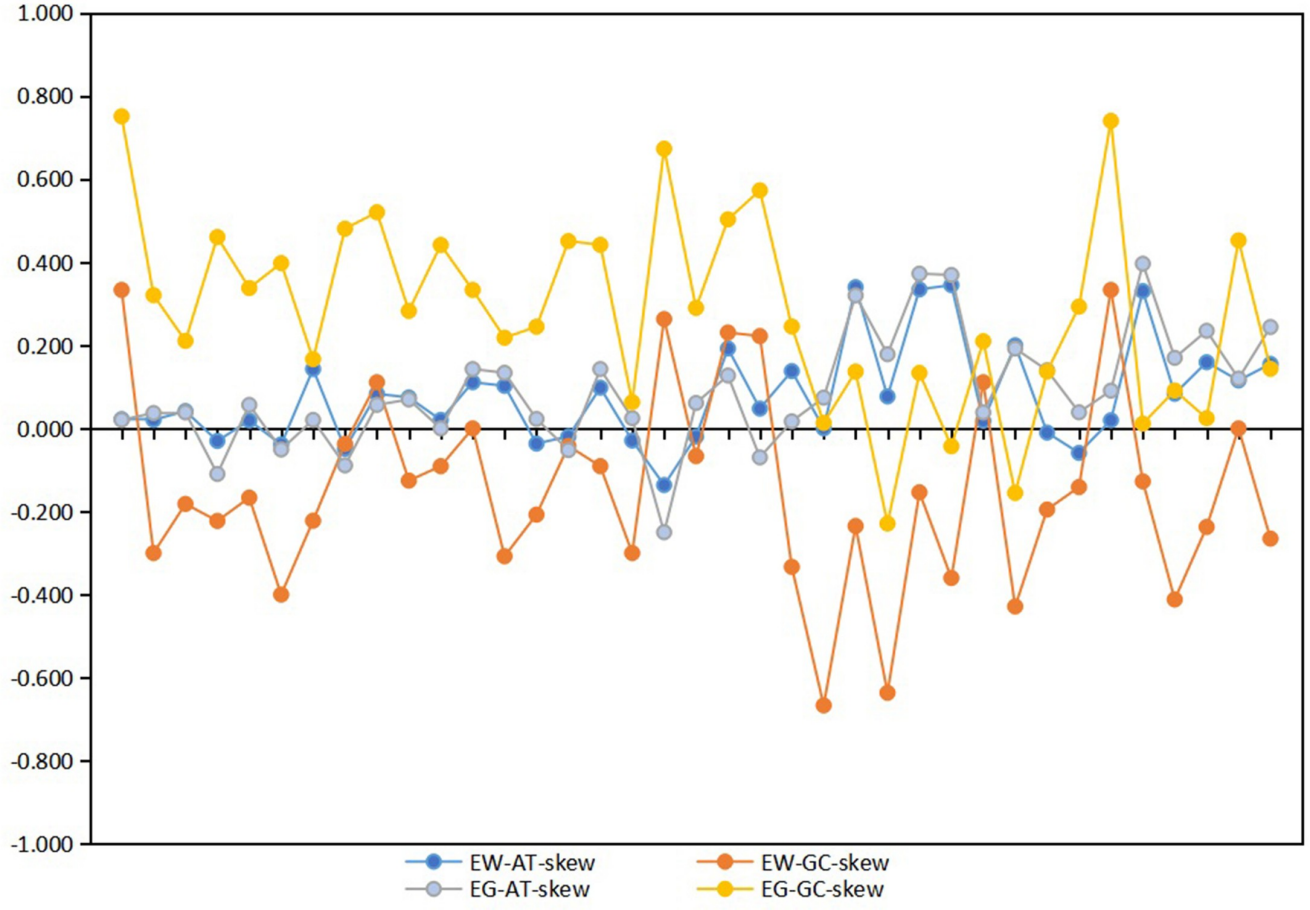
AT and GC skews values for the 37 mitochondrial genome of *E*. *wengangensis* and *E*. *gracilis*. Each point indicates an individual gene.

The AT contents and skew statistics are shown in Table 4. The mitochondrial genomes of *E*.*wengangensi*s and *E*. *gracilis* exhibit heavy AT nucleotide bias, with A + T% for the whole sequence 76.6% and 77.0%, respectively. Similar patterns of nucleotide composition are also found in other leafhopper species [38, 47]. The control region (CR) has the strongest A + T% bias, while the PCGs shows the lowest A + T% among whole genes. The whole genome has positive AT-skews (0.015, 0.140) and negative GC-skews (-0.154, -0.157). Analysis of 37 individual genes of the two species shows that AT-skews are mostly positive, while for GC-skews, the genes of *E*. *wengangensis* are mostly negative, but *E*. *gracilis* are mostly positive (Fig 2 and S2 Table). Positive AT-skews indicates that the content of base A is higher than that of base T. However, although the AT-skews is negative in a few genes, the difference in absolute value was minimal. For GC-skews, a negative value indicates that the content of base G is lower than that of base C, while a positive value is an opposite. Overall, the base composition of these two species is skewed toward A and C.

**Table 4:** Nucleotide compositions, AT- skew and GC-skew in different regions of *E*. *wengangensis* and *E*. *gracilis* mitochondrial genome.

*E*. *wengangensis*								
Feature	A%	C%	G%	T%	A+T%	AT-skew	GC-skew	Length(bp)
Whole	42.7	13.5	9.9	33.9	76.6	0.115	-0.154	14830
PCGs	42.2	14.2	10.5	33.2	75.4	0.119	-0.150	10964
1st codon position	42.5	14.8	13.3	29.5	72.0	0.181	-0.053	3655
2nd codon position	38.1	16.4	10.3	35.3	73.4	0.038	-0.228	3655
3rd codon position	46.0	11.3	7.8	34.8	80.8	0.139	-0.183	3654
tRNA	41.0	12.3	10.2	36.5	77.5	0.058	-0.093	1429
*16S*	47.5	11.2	6.9	34.4	81.9	0.159	-0.237	1188
*12S*	46.1	12.8	7.4	33.7	79.8	0.155	-0.265	727
CR	42.9	8.6	8.4	40.2	83.1	0.032	-0.012	525
***E*. *gracilis***								
Whole	43.9	13.3	9.7	33.1	77.0	0.140	-0.157	14627
PCGs	42.9	14.1	10.3	32.7	75.6	0.134	-0.144	10976
1st codon position	42.7	15.4	13.0	28.9	71.6	0.193	-0.085	3659
2nd codon position	40.3	15.6	10.0	34.2	74.5	0.082	-0.219	3659
3rd codon position	45.8	11.3	7.8	35.1	80.9	0.132	-0.183	3658
tRNA	41.6	11.3	10.0	37.1	78.7	0.057	-0.061	1461
*16S*	51.2	11.1	6.0	31.7	82.9	0.235	0.024	1202
*12S*	49.9	11.8	7.9	30.3	80.3	0.244	0.144	735
CR	43.9	5.7	3.3	47.2	91.1	-0.036	-0.267	212

### Protein-coding genes and codon usage

Among the 13 PCGs of 56 species, The average AT content values of PCGs is 76.55%, Iassinae is the highest at 79.57%, and Ledrinae is the lowest at 74.36%. The third codon of 42 species is higher than the first codon. Moreover, 14 are the opposite. The range of AT-skews between subfamilies is -0.2745~0.1680, and the GC-skews is -0.2337~0.1589. Coelidiinae has the highest AT-skews and the lowest GC-skews, while Ledrinae is the opposite. The AT-skews of 13 species are all negative, which from Empoascini in Typhlocybinae, Idiocerinae, Cicadellinae, Ledrinae, and Evacanthinae. Furthermore, the other subfamilies and species are all positive. In GC-skews, only Ledrinae and six species have positive values, and the others are negative (Table 5 and S3 Table). All 62 available codons are used in 11 subfamilies. Synonymous codon usage bias was observed in 56 mitochondrial genomes, and UUA (Leu), UCG (Ser), AUU (Ile), AUA (Met), *etc*. are the most commonly used codons in many species.

**Table 5:** 13PCGs nucleotide compositions, AT- skews and GC-skews in 11 subfamilies of Cicadellidae.

Subfamily	A+T%	AT-skew	GC-skew	Pos1	Pos2	Pos3
Typhlocybinae	76.22	0.0799	-0.0947	76.12	73.96	78.58
Empoascini (tribe)	77.38	**-0.0625**	0.0035	78.12	75.71	78.27
Other tribes	75.84	0.1274	-0.1275	75.46	73.38	78.69
Deltocephalinae	75.55	0.1115	-0.1618	74.14	73.54	78.96
Idiocerinae	76.99	**-0.0985**	-0.0056	76.09	75.60	79.28
Iassinae	79.57	0.1592	-0.1509	77.03	79.55	82.13
Cicadellinae	76.37	**-0.0690**	-0.0238	78.96	73.08	77.06
Coelidiinae	76.69	0.1680	-0.2337	77.56	74.30	78.19
Megophthalminae	76.26	0.1627	-0.1922	77.35	76.16	75.27
Mileewinae	78.56	0.1074	-0.1847	71.15	79.17	85.35
Macropsinae	75.93	0.1559	-0.1134	77.12	70.85	79.83
Ledrinae	74.36	**-0.2745**	0.1589	73.53	71.21	78.34
Evacanthinae	78.74	**-0.0115**	-0.0402	79.93	73.71	82.58

Located on the major strand (H-strand), while the other four PCGs are located on the minor strand (L-strand). The average AT content values of PCGs are 75.4% and 75.6% in *E*. *wengangensis* and *E*. *gracilis*, respectively, and the third codon position (80.8%, 80.9%) has an AT content much higher than that of the first (72.0%, 71.6%) and second (73.4%, 74.5%) positions. AT-skews of all codon positions are positive, while GC-skews are negative. All 13 PCGs have the standard ATN as the start codon, while *nad5* and *atp8* genes have TTG, a pattern also observed in other leafhopper mitogenomes [25, 26]. Conventional stop codons (TAA or TAG) appear in 11 PCGs, except that *cox2* and *nad5* use an incomplete codon (a single T—) as the stop codon (Tables 3 and 4).

**Fig 3:**
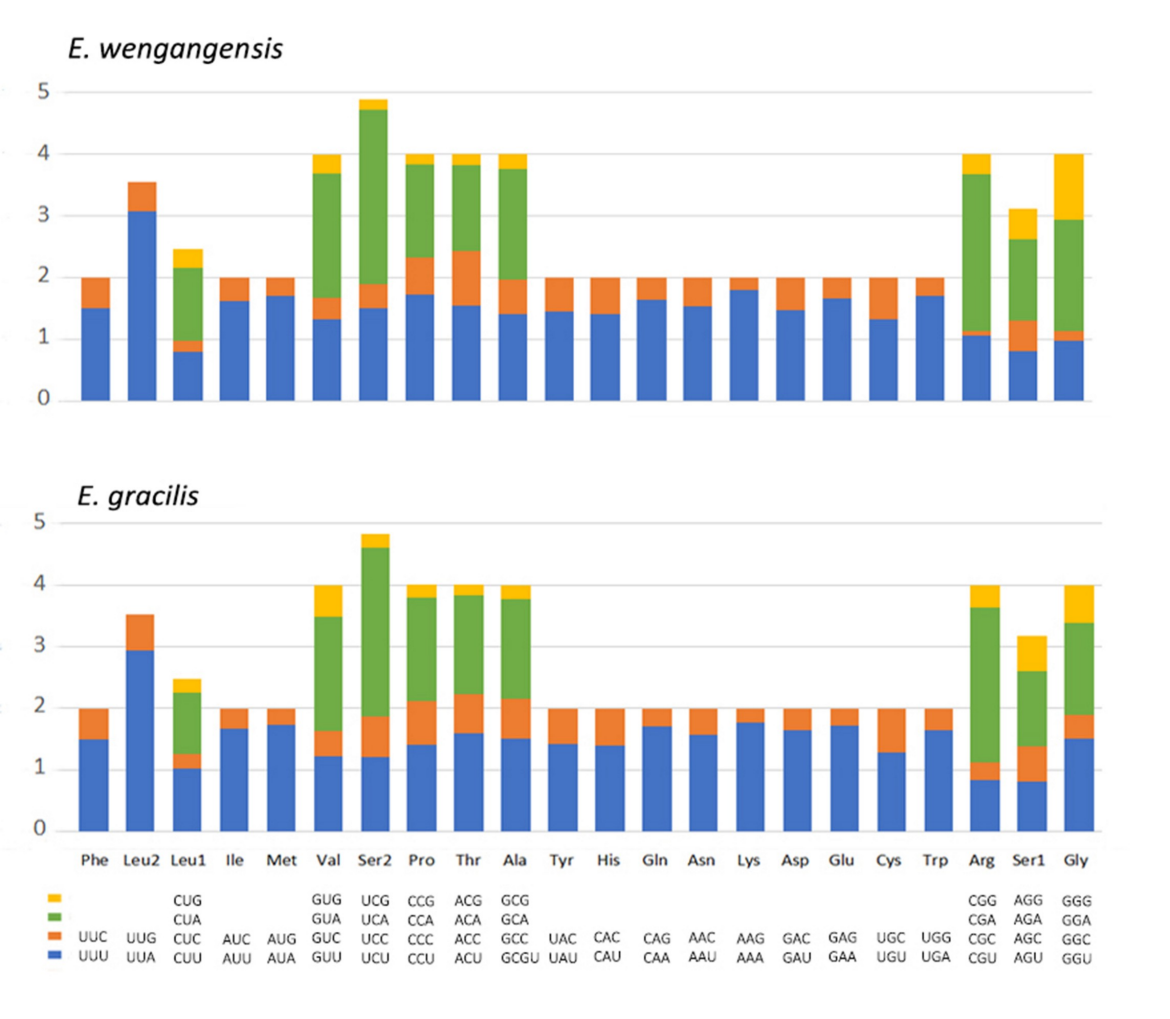
Relative Synonymous Codon Usage (RSCU) of mitochondrial genomes for *E*. *wengangensis* and *E*. *gracilis*.

The relative synonymous codon usage (RSCU) was calculated and summarized, and All 62 available codons (excluding TAA and TAG) are used in *E*. *wengangensis* and *E*. *gracilis*. After excluding the stop codons, these two species have 3654 and 3658 PCG codons, respectively (Fig 3 and Table 6). Synonymous codon usage bias was observed in both mitochondrial genomes, and 22 codons are used more frequently than other codons. The four most abundant codons are UUA (Leu), UCG (Ser), GUA (Val) and GAA (Glu), and the least used codons were CGC (Arg) and ACG (Thr). The preferred codons all end with A or U, thus resulting in a strong A + T bias at the third codon position.

**Table 6:** Codon and relative synonymous codon usage (RSCU) of 13 PCGs in the mt genomes of *E*. *wengangesis* and *E*. *gracilis*.

Amino Acid	*Codon*	*Count/RSCU*				Amino Acid	*Codon*	*Count/RSCU*			
		*E*. *wengangesis*		*E*. *gracilis*				*E*. *wengangesis*		*E*. *gracilis*	
Phe	UUU	**161**	**1.5**	**141**	**1.5**	Tyr	UAU	**117**	**1.45**	**111**	**1.42**
	UUC	53	0.5	47	0.5		UAC	44	0.55	45	0.58
Leu2	UUA	**206**^**a**^	**3.07**	**196**	**2.94**	His	CAU	**53**	**1.41**	**63**	**1.4**
	UUG	32	0.48	39	0.58		CAC	22	0.59	27	0.6
Leu1	CUU	**54**	**0.8**	**68**	**1.02**	Gln	CAA	**90**	**1.64**	**110**	**1.71**
	CUC	12	0.18	16	0.24		CAG	20	0.36	19	0.29
	CUA	79	1.18	66	0.99	Asn	AAU	**240**	**1.54**	**261**	**1.57**
	CUG	20	0.3	15	0.23		AAC	72	0.46	71	0.43
Ile	AUU	**257**	**1.62**	**263**	**1.67**	Lys	AAA	**329**	**1.8**	**324**	**1.77**
	AUC	61	0.38	52	0.33		AAG	36	0.2	43	0.23
Met	AUA	**211**	**1.7**	**206**	**1.73**	Asp	GAU	**44**	**1.47**	**56**	**1.65**
	AUG	37	0.3	32	0.27		GAC	16	0.53	12	0.35
Val	GUU	35	1.33	33	1.22	Glu	GAA	**111**	**1.66**	**111**	**1.72**
	GUC	9	0.34	11	0.41		GAG	23	0.34	18	0.28
	GUA	**53**	**2.02**	**50**	**1.85**	Cys	UGU	**24**	**1.33**	**18**	**1.29**
	GUG	8	0.3	14	0.52		UGC	12	0.67	10	0.71
Ser2	UCU	54	1.5	42	1.21	Trp	UGA	**65**	**1.71**	**67**	**1.65**
	UCC	14	0.39	23	0.66		UGG	11	0.29	14	0.35
	UCA	**102**	**2.83**	**95**	**2.73**	Arg	CGU	13	1.06	9	0.84
	UCG	6	0.17	8	0.23		CGC	1	0.08	3	0.28
Pro	CCU	**61**	**1.73**	**48**	**1.41**		CGA	**31**	**2.53**	**27**	**2.51**
	CCC	21	0.6	24	0.71		CGG	4	0.33	4	0.37
	CCA	53	1.5	57	1.68	Ser1	AGU	29	0.81	28	0.81
	CCG	6	0.17	7	0.21		AGC	18	0.5	20	0.58
Thr	ACU	**76**	**1.55**	**79**	**1.6**		AGA	**47**	**1.31**	**42**	**1.21**
	ACC	43	0.88	31	0.63		AGG	18	0.5	20	0.58
	ACA	68	1.39	79	1.6	Gly	GGU	25	0.98	39	1.51
	ACG	9	0.18	9	0.18		GGC	4	0.16	10	0.39
Ala	GCU	30	1.41	26	1.51		GGA	**46**	**1.8**	**38**	**1.48**
	GCC	12	0.56	11	0.64		GGG	27	1.06	16	0.62
	GCA	**38**	**1.79**	**28**	**1.62**	*	UAA	152	1.73	179	1.77
	GCG	5	0.24	4	0.23		UAG	24	0.27	23	0.23

^a^The higher values of preferentially used codons are in bold.

### Transfer RNA and ribosomal RNA genes

The predicted tRNA length of 56 species is between 60~75bp, all of which are the typical clover-leaf secondary structure while only *trnS1* lacks the dihydrouridine (DHU) stem and forms a simple loop. Furthermore, mismatches such as GU, AA, *etc*. often occur in tRNA. The structures and characteristics of *16S* and *12S* are similar in each subfamily. The sizes are 1038~1426bp, 707~789bp, with an average of 1188bp and 740bp, respectively. The AT content of *16S* is higher than that of *12S*. Deltocephalinae and Coelidiinae are the largest of *16S* and *12S*, respectively. Whole nucleotide compositions, AT- skew and GC-skew in 11 subfamilies of Cicadellidae.

All 22 typical tRNA genes are present in the *E*. *wengangensis* and *E*. *gracilis* mitochondrial genomes, fourteen genes are oriented on the major strand (H-strand), whereas the others are transcribed on the minor strand (L-strand). Their nucleotide lengths are almost identical between species, ranging from 62 (*trnG* and *trnT*) bp to 71 bp (*trnK* and *trnM*) (Table 3). The average AT content values of tRNAs are 77.5% and 78.7%, respectively, and the tRNA genes have negligible AT and GC-skews (Table 4).

**Fig 4:**
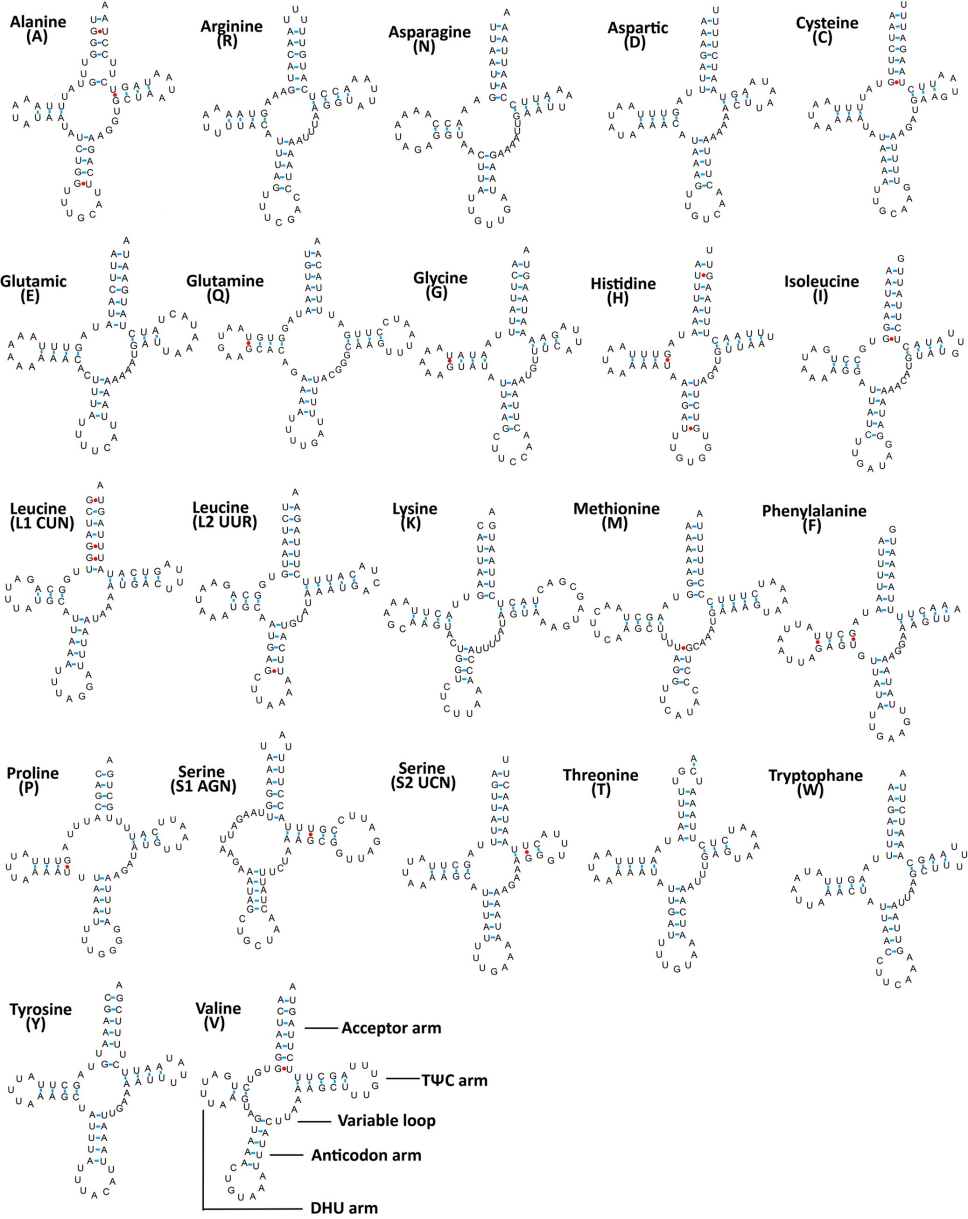
Inferred secondary structures of 22 tRNAs from *E*. *wengangensis*. Watson-Crick base pairings are illustrated by lines (-), whereas GU base pairings are illustrated by red dots. Structural elements in tRNA arms and loops are illustrated as for *trnV*.

Based on the secondary structure, a total of 20 and 21 G-U weak base pairs are found in *E*. *wengangensis* and *E*. *gracilis* of tRNAs respectively (Fig 4 and S1 Fig), forming weak bonds and located in AA stems (11bp), T stems (3 and 2bp) and DHU stems (6 and 8 bp). Most mismatched nucleotides are G-U pairs, which form weak bonds in tRNA and non-classical pairs in tRNA secondary structure, similar to other Cicadellidae [40, 46].

Leafhopper ribosomal RNA (rRNA) includes *16S* RNA and *12S* RNA. These two genes are highly conserved and are encoded on the minor strand (L-strand). Similar to other known insects, the content of A + T% in *16S* is higher than that of *12S*. The *16s* genes of *E*. *wengangensis* and *E*. *gracilis* are 1188bp and 1202bp in length, with AT content of 81.90% and 82.90%, respectively, and located between *trnL2* and *trnV*. The *12S* rRNA genes of both are 727bp and 735 bp in length, with AT contents of 79.80% and 80.30%, respectively, and located after *trnV*. The rRNA genes showed a positive AT-skew and GC-skew (Table 4). These features are similar to those observed in other insects [49, 69, 70].

### Control region

**Fig 5:**
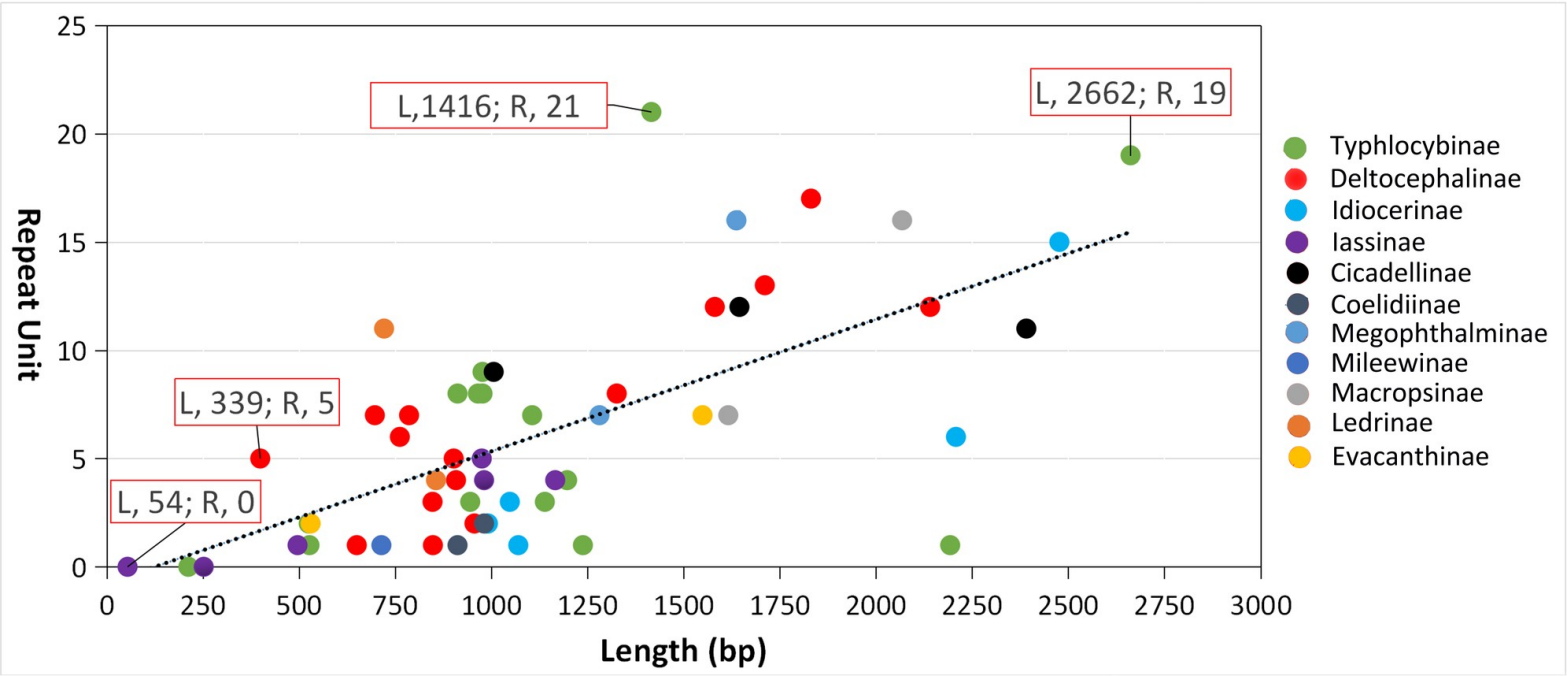
Correlation between the size of CR regions and repeat units in 56 species.

The CR regions of 11 subfamilies range from 54 to 2662 bp, with an average of 1142 bp, the smallest in Iassinae and the largest in Typhlocybinae. The AT content is higher which ranging from 79.35% to 88.08%, with an average of 84.73%, and Mileewinae is the highest while Evacanthinae lowest. Among the 56 species, the repeat units range from 0 to 21. The smallest repeat unit from Typhlocybinae and Iassinae, and the largest repeat unit from Typhlocybinae. Spss 22.0 software was used to analyze the correlation between CR region size and repeat unit in 56 species. The results showed that there was a significant correlation between them (R = 0.672, P < 0.05), and the repeat units of most Cicadellidae insects were positively correlated with the size of CR regions (Fig 5).

**Fig 6:**
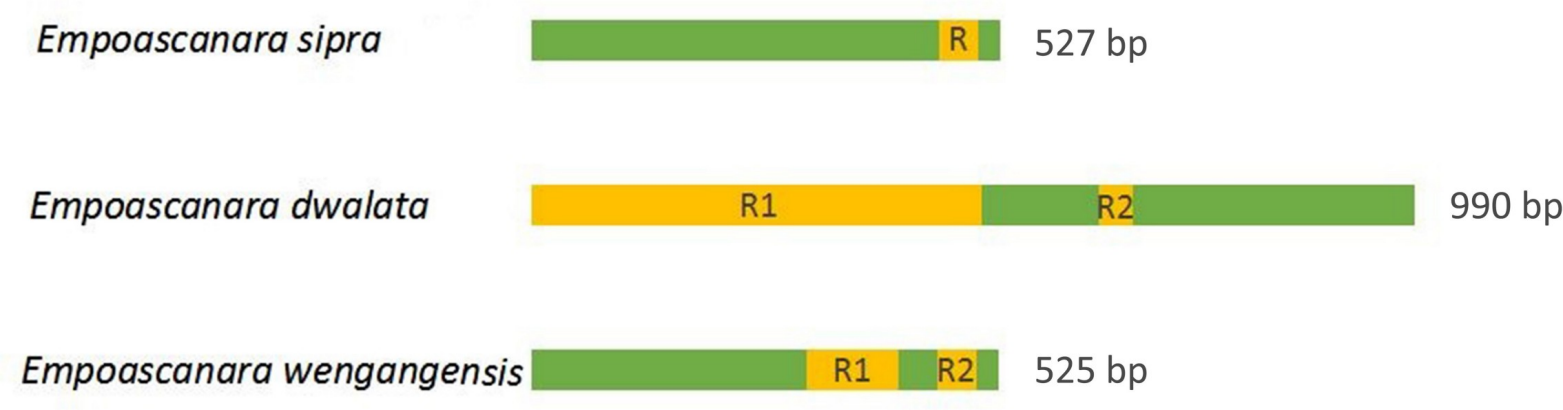
Organization of the control region structure in the mitochondrial genomes of three *Empoascanara* species. R: repeat unit.

Like the typical insect mitochondrial genome, the mt genomes of *E*. *wengangensis* and *E*. *gracilis* have a sizeable non-coding region identified as the control region and located downstream of *12S*. Control regions of both species are rich in AT, their lengths are 525bp and 212bp, and the AT contents are 83.1% and 91.1%, respectively (Table 4). The control regions in the four available *Empoascanara* mitogenomes are various and not highly conserved, and their lengths range between 212 and 990 bp with variable numbers of repeat sequences (Fig 6). No tandem repeat units were found in *E*. *gracilis*; *E*. *sipra* includes one type of repeat unit (R); two kinds of repeats (R1, R2) are found in *E*. *dwalata* and *E*. *wengangensis* with various lengths and copy numbers.

### Nucleotide diversity and evolutionary rate analysis

**Fig 7:**
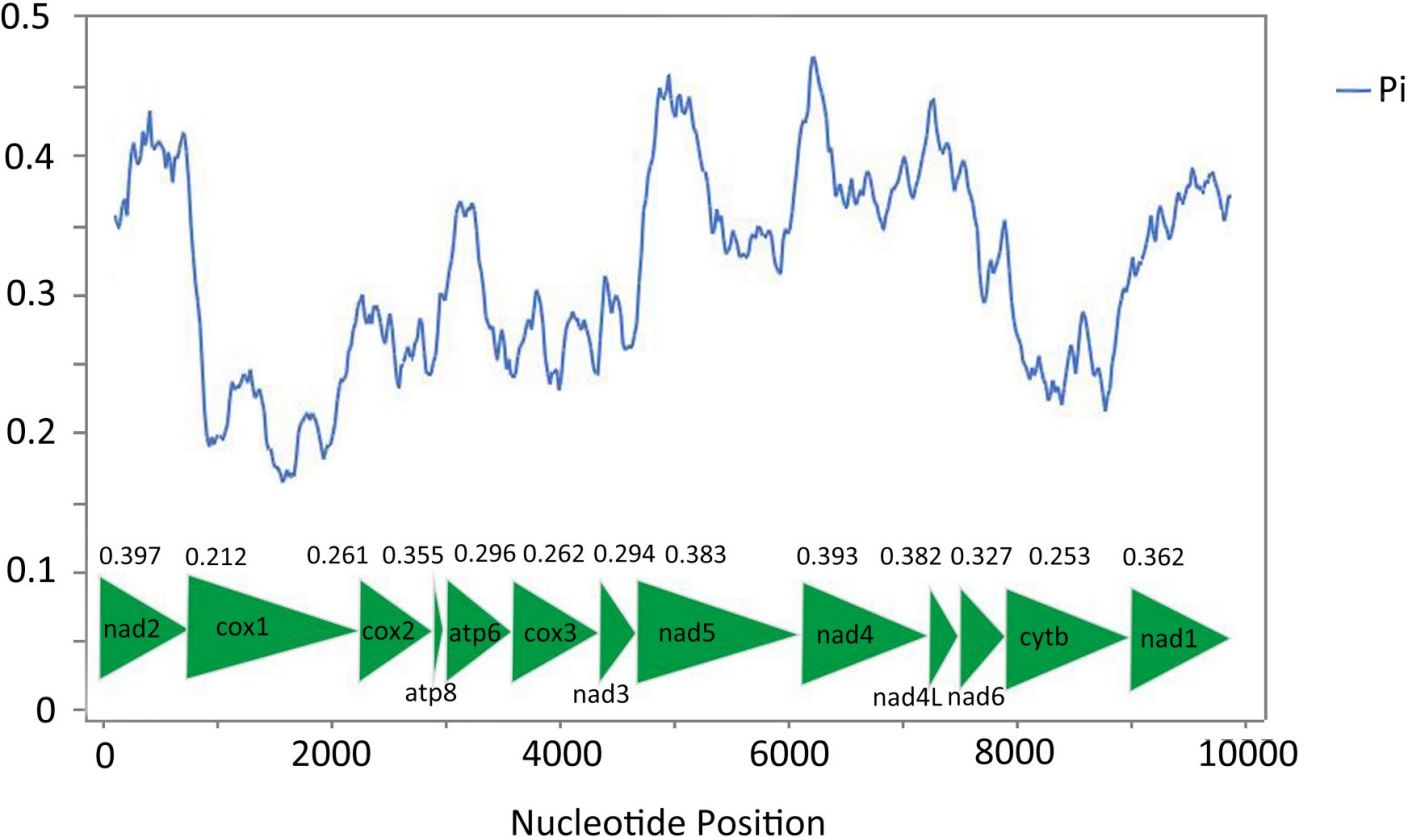
Nucleotide diversities and sliding window analysis of 13 PCGs of the 56 Cicadellidae species. The blue curve shows the value of nucleotide diversity (Pi). Pi value of each PCG was shown above the arrows.

**Fig 8:**
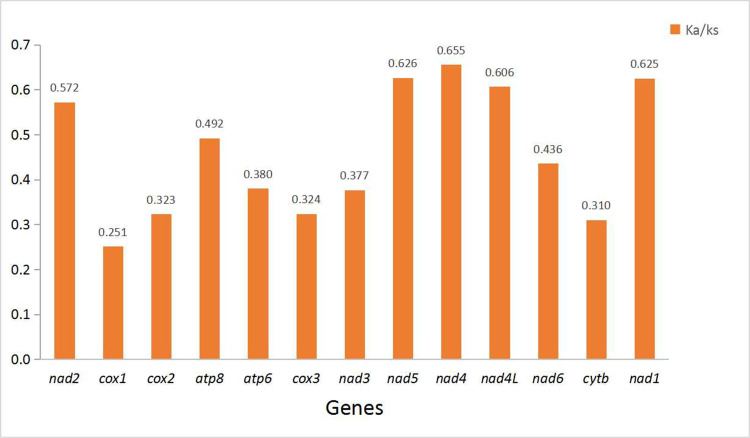
The ratio of non-synonymous (Ka) to synonymous (Ks) substitution rates of each 13 PCGs among 56 Cicadellidae species.

The sliding window analysis shows highly variable nucleotide diversity (Pi values) among 13 PCGs sequences of the 56 mitogenomes (Fig 7). The genes *nad2*, *nad4*, *nad4L* and *nad5* have high nucleotide diversity of 0.397, 0.393, 0.382, and 0.380, respectively, while the genes *cox3*, *cox2*, *cytb* and *cox1* have comparatively low nucleotide diversity of 0.262, 0.261, 0.253 and 0.212 respectively. The pairwise Ka/Ks analysis shows that the average Ka/Ks ratios (ω) of 13 PCGs ranged from 0.251 to 0.655 (0 < ω < 1) (Fig 8), indicating that these genes are under purifying selection [71]. The genes *nad4*, *nad5*, *nad1* and *nad6* exhibit comparatively high Ka/Ks ratios of 0.655, 0.626, 0.625 and 0.606, while the values of *cox3*, *cox2*, *cytb* and *cox1* were relatively low, respectively 0.324, 0.323, 0.310 and 0.251.

Nucleotide diversity analysis are primary for identifying the regions with large nucleotide divergence, especially useful for designing species-specific markers [72, 73]. These are useful for taxa with highly variable morphological characteristics, especially Cicadellidae species. For a long time, gene *cox1* has been considered as a universal barcode for identifying animal species [74], but in these 56 Cicadellidae species, it is the slowest evolving and least changing gene among 13 PCGs. If it is proved that the resolution of *cox1* is very low in 13 PCGs, then other genes with sufficient large size, rapid evolution and high Ka/Ks ratio can be used as potential molecular markers in population genetics [73, 75]. However, Part of the value of *cox1* as a marker is also the availability of conserved primer sites and relatively low AT% bias. In this case, *nad4* and *nad5* may be evaluated as potential DNA markers that define the Cicadellidae insect species, but the result needs to be verified by multiple parties.

### Phylogenetic relationships

**Fig 9:**
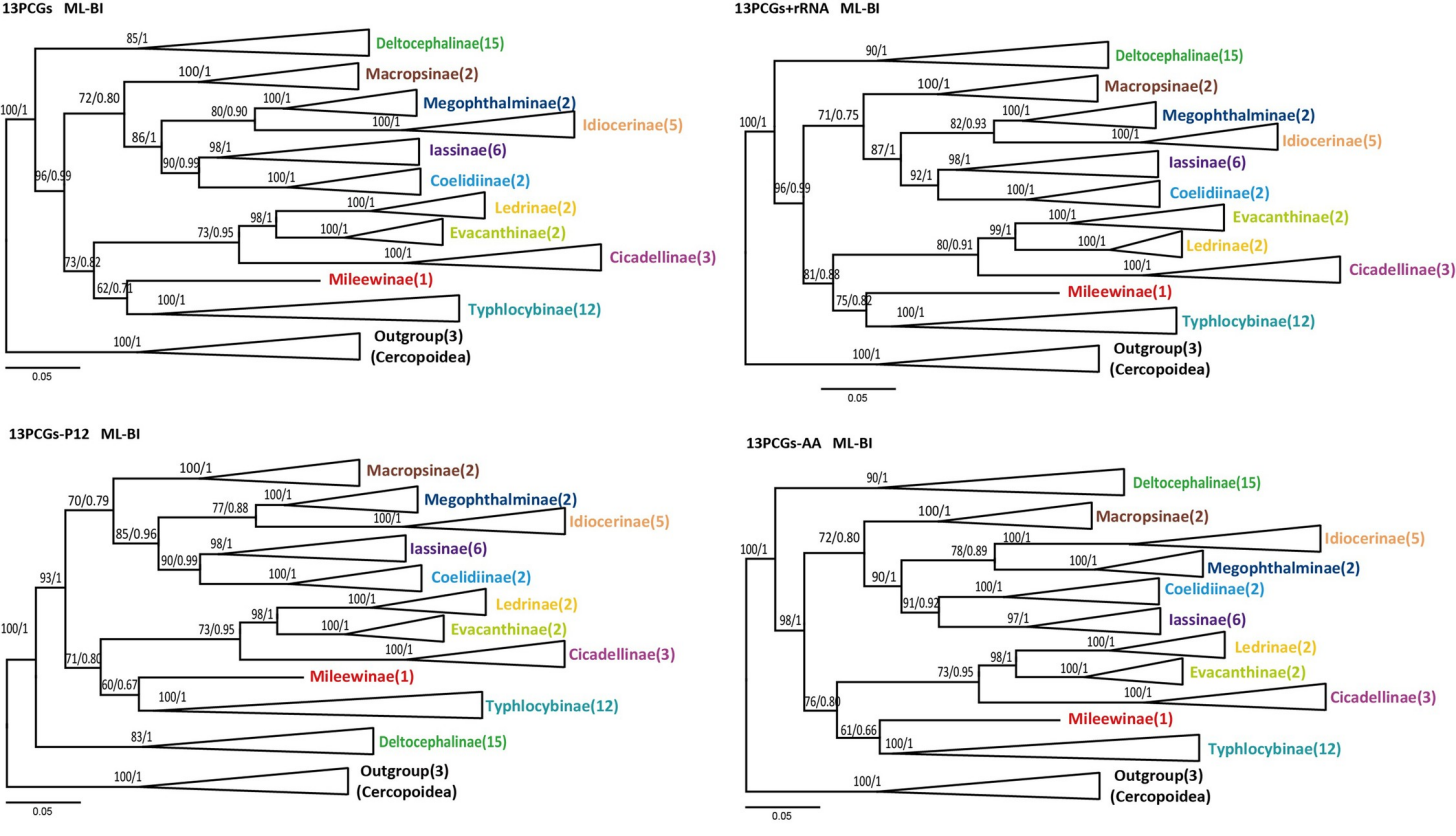
Phylogenetic trees of Cicadellidae inferred by maximum likelihood (ML) and Bayesian (BI) methods based on protein-coding genes.

**Fig 10:**
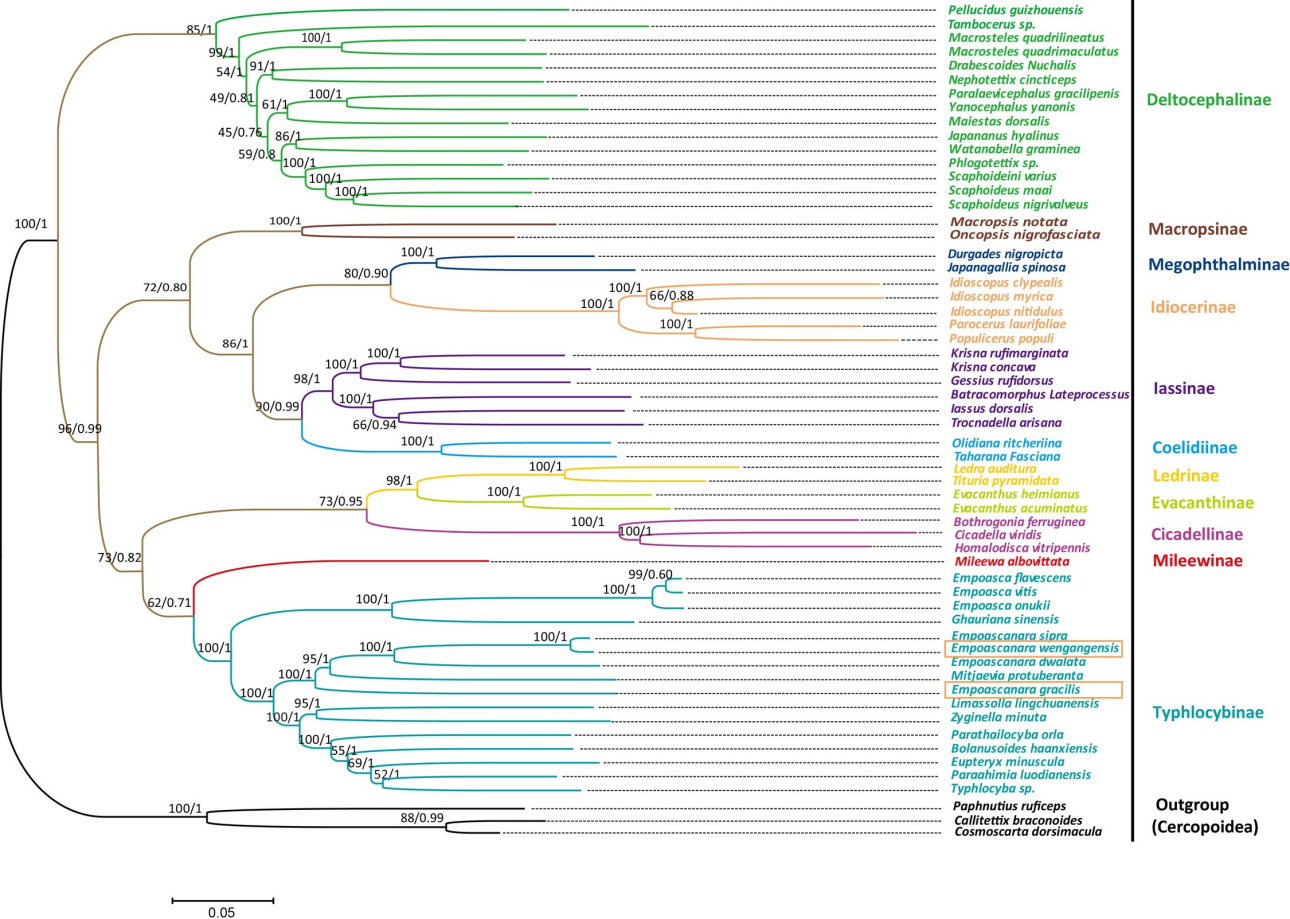
ML and BI Phylogenetic tree inferred from 13 PCGs of Cicadellidae. The first number at each node is bootstrap proportion (BP) of maximum likelihood (ML) analyses, and the second number is Bayesian (BI) posterior probability (PP).

Cicadellidae subfamilies’ relationship has always attracted related scholars’ attention because of the small size, and the morphological characteristics are difficult to distinguish. Early scholars made preliminary explorations of phylogenetic relationships based on morphological characteristics, and then analyzed them with molecular data. However, most studies are based on gene fragments, and data are relatively lacking, so the results do not reflect the relationship between subfamilies very well, and more data are needed for verification [24, 28, 46, 76, 77]. In this study, the results are the same as other recent studies of the mitochondrial genome (Figs 9 and 10). Most currently recognized leafhopper subfamilies were recovered as monophyletic but relationships among some subfamilies are not well resolved. And compared with the phylogenetic tree constructed using nuclear genes (*28S*), the relationship between the subfamilies is partly different, for example, Cicadellinae and Coelidiinae are sister groups, but they are not grouped together in this study. This may be because the study used only one nuclear gene fragment and the amount of data was insufficient [17]. At present, the most researched based on the complete sequence data are the horned leafhoppers of Cicadellidae, and other groups are rarely reported or not. Many studies have shown that Deltocephalinae species constituted one clade and tended to be placed at the tree’s basal position as the sister group to the other leafhoppers, as in this study. The relationship between Mileewinae and its related subfamilies has also been studied. Young transferred Mileewanini from Cicadellinae to Typhlocybinae in 1965, thinking that Mileewanini has more similarities with Typhlocybinae. Mahmood questioned this view and moved the tribe back to Cicadellinae in related work. Later [78], Young suggested Mileewanini as a separate subfamily in 1968 [79], and it has been adopted by many scholars in recent years [6, 19, 21]. In 2019, He Hongli used PCGs data to conduct a preliminary phylogenetic study on this subfamily’s taxonomic status. The results show that Mileewinae is closer to Typhlocybinae than Cicadellinae and similar to Dietrich morphological phylogeny research [80]. Our research also shows that Mileewinae is a monophyletic group and is a sister group with Typhlocybinae. However, this result is only based on a species of Mileewinae, and more molecular data of this subfamily are needed to confirm this conclusion. Similarly, Evacanthinae’s kinship issue has yet to be resolved. Although Dietrich and Wang Yang (2017) combined morphological and molecular data to conduct phylogenetic analysis, their relationship with closely related groups has not been clearly reconstructed [6, 17, 81]. The sister group of Evacanthinae may be Coelidiinae + Neocoelidiinae, Mileewinae + Typhlocybinae, Coelidiinae + Mileewinae + Signoretiinae, or Cicadellinae + Mileewinae. This paper shows that the sister group of Evacanthinae is Ledrinae + Cicadellinae. And phylogenetic analysis grouped the two newly sequenced species (*E*. *wengangensis* and *E*. *gracilis*) with other species of Typhlocybinae, which was separated from other subfamilies, and all Erythroneurini insects gathered together. However, *E*. *gracilis* grouped into a single group, not grouped with species of the same genus (*Empoascanara*). This result does not match the traditional classification, and other nuclear genes or transcriptome genes may be needed to verify the result. Based on the above research, although mitochondrial genes are now widely used in phylogenetic analysis, but the results obtained by different gene fragment combinations will have certain differences. The amount of data and the model have a great influence on the accuracy of the research results. Perhaps we need to combine other data to analyze the mitochondrial genome in a deeper level.

## Conclusion

This paper analyzes and summarizes the complete mitochondrial genome structure characteristics of 11 leafhopper subfamilies and two newly sequenced Typhlocybinae species, *E*. *wengangensis* and *E*. *gracilis*, and analyzes the elemental composition, location, secondary structure and other characteristics of PCGs, tRNA genes, rRNA genes and control regions. Furthermore, using 13PCGs and rRNA data to analyzes the nucleotide diversity, evolution rate, and the phylogenetic relationship between the subfamilies of 56 species, verifying the taxonomic status analysis of *E*. *wengangensis* and *E*. *gracilis*. The analysis results show that the genome structures of the subfamilies and the newly sequenced two species are very similar, and the size of the CR region is significantly related to the repeat unit. However, in the entire AT-skew and CG-skew, the other subfamilies AT-skew are all positive, and CG-skew are negative, while Empoascini of Typhlocybinae and Ledrinae are the opposite. Moreover, among 13PCGs, the AT-skews of 13 species are all negative while CG-skews are positive, which from Empoascini in Typhlocybinae, Idiocerinae, Cicadellinae, Ledrinae, and Evacanthinae. This feature is consistent with the phylogenetic relationship displayed by the phylogenetic tree. We speculate that AT-skew and CG-skew have specific indications for the phylogeny of Cicadellidae species.

Phylogenetic analysis shows that ML and PB analysis produce almost consistent topologies between different data sets and models, and some relationships are highly supported and remain unchanged. Mileewinae is a monophyletic group and is a sister group with Typhlocybinae, and the sister group of Evacanthinae is Ledrinae + Cicadellinae. And phylogenetic analysis grouped the two newly sequenced species (*E*. *wengangensis* and *E*. *gracilis*) with other species of Typhlocybinae, which was separated from other subfamilies, and all Erythroneurini insects gathered together. However, *E*. *gracilis* grouped into a single group, not grouped with species of the same genus (*Empoascanara*). This result does not match the traditional classification, and other nuclear genes or transcriptome genes may be needed to verify the result. Nucleotide diversity analysis shows that *nad4* and *nad5* may be evaluated as potential DNA markers defining the Cicadellidae insect species. This study confirms the results of previous studies indicating that mitochondrial genome sequences are informative of leafhopper phylogeny. The new data provided here will facilitate future comparative studies of leafhopper mitogenomes and accentuate the need for more comparative data. However, more data is still needed to verify the above research results, especially for the subfamilies, whose taxonomic status is disputed. At present, there are little or no sequencing data.

## Supporting information

S1 Fig Inferred secondary structures of 22 tRNAs from *E*. *gracilis*. Watson-Crick base pairings are illustrated by lines (-), whereas GU base pairings are illustrated by red dots. Structural elements in tRNA arms and loops are illustrated as for trnV.
(DOCX)

S1 Table Whole nucleotide compositions, AT- skews and GC-skews in 56 species of Cicadellidae. (DOCX)

S2 Table Nucleotide composition, AT and GC skews calculated for the 37 mitochondrial genome of *E*. *wengangensis* and *E*. *gracilis*. (DOCX)

S3 Table 13 PCGs nucleotide compositions, AT- skews and GC-skews in 56 species of Cicadellidae. (DOCX)

S4 Table Codon and relative synonymous codon usage (RSCU) of 13 PCGs in the mt genomes of *E*. *wengangensis* and *E*. *gracilis*. (DOCX)
