# Supplementary material for: Structural features and phylogenetic implications of Cicadellidae subfamily and two new mitogenomes leafhoppers
Source: PLoS One. 2021 May 14;16(5):e0251207. doi: 10.1371/journal.pone.0251207 (PMC8121325; doi:10.1371/journal.pone.0251207)

**S1 Fig. Inferred secondary structures of 22 tRNAs from *E. gracilis*.** Watson-Crick base pairings are illustrated by lines (-), whereas GU base pairings are illustrated by red dots. Structural elements in tRNA arms and loops are illustrated as for trnV.


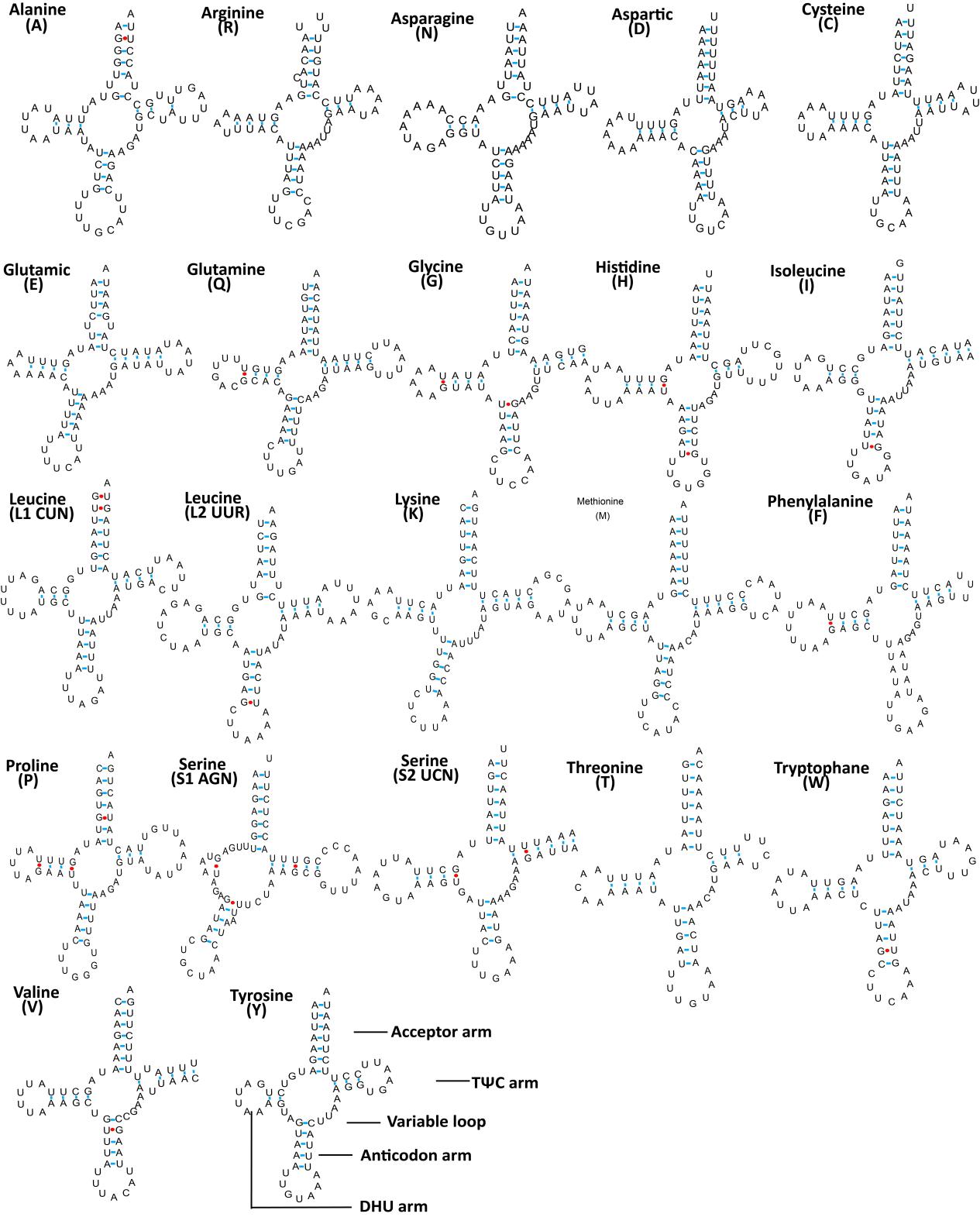

Supplement: S1 Fig — Watson-Crick base pairings are illustrated by lines (-), whereas GU base pairings are illustrated by red dots. Structural elements in tRNA arms and loops are illustrated as for trnV. (DOCX) [file pone.0251207.s001.docx]
