# Supplementary material for: Structural features and phylogenetic implications of Cicadellidae subfamily and two new mitogenomes leafhoppers
Source: PLoS One. 2021 May 14;16(5):e0251207. doi: 10.1371/journal.pone.0251207 (PMC8121325; doi:10.1371/journal.pone.0251207)
Supplement: S1 Table — (DOCX) [file pone.0251207.s002.docx]

**S1 Table. Whole nucleotide compositions, AT- skews and GC-skews in 56 species of Cicadellidae.**

| **Subfamily** | **Specie** | **Length(bp)** | **A** | **C** | **G** | **T(U)** | **A+T%** | **AT-skew** | **GC-skew** |
| --- | --- | --- | --- | --- | --- | --- | --- | --- | --- |
| Typhlocybinae | *Empoascanara wengangensis* | 14830 | 42.70 | 13.50 | 9.90 | 33.90 | 76.60 | 0.1149 | -0.1538 |
|  | *Empoascanara gracilis* | 14627 | 43.90 | 13.30 | 9.70 | 33.10 | 77.00 | 0.1403 | -0.1565 |
|  | *Empoascanara dwalata* | 15271 | 41.65 | 12.98 | 10.63 | 34.74 | 76.39 | 0.0905 | -0.0996 |
|  | *Empoascanara sipra* | 14827 | 42.66 | 13.41 | 9.98 | 33.95 | 76.61 | 0.1137 | -0.1465 |
|  | *Mitjaevia protuberanta* | 15472 | 40.01 | 11.92 | 10.63 | 37.43 | 77.44 | 0.0334 | -0.0573 |
|  | *Limassolla lingchuanensis* | 15716 | 43.18 | 11.63 | 9.56 | 35.64 | 78.82 | 0.0957 | -0.0976 |
|  | *Paraahimia luodianensis* | 16497 | 46.18 | 11.63 | 8.38 | 33.81 | 80.00 | 0.1547 | -0.1624 |
|  | *Zyginella minuta* | 15544 | 42.96 | 13.54 | 10.25 | 33.25 | 76.21 | 0.1273 | -0.1385 |
|  | *Parathailocyba orla* | 15382 | 45.77 | 12.26 | 8.67 | 33.31 | 79.07 | 0.1576 | -0.1718 |
|  | *Typhlocyba sp.* | 15223 | 43.85 | 13.05 | 9.89 | 33.21 | 77.06 | 0.1382 | -0.1380 |
|  | *Eupteryx minuscula* | 16944 | 43.60 | 11.36 | 9.87 | 35.17 | 78.78 | 0.1070 | -0.0701 |
|  | *Bolanusoides shaanxiensis* | 15274 | 45.72 | 11.94 | 9.09 | 33.24 | 78.96 | 0.1581 | -0.1354 |
|  | *Empoasca vitis* | 15154 | 38.16 | 11.19 | 10.48 | 40.17 | 78.34 | **-0.0257** | **-0.0326** |
|  | *Ghauriana sinensis* | 15491 | 38.69 | 10.43 | 10.02 | 40.87 | 79.56 | **-0.0274** | **-0.0199** |
|  | *Empoasca flavescens* | 15152 | 38.08 | 11.12 | 10.52 | 40.28 | 78.36 | **-0.0281** | **-0.0281** |
|  | *Empoasca onukii* | 15167 | 38.13 | 11.19 | 10.50 | 40.19 | 78.31 | **-0.0263** | **-0.0319** |
| Deltocephalinae | *Pellucidus guizhouensis sp.* | 16555 | 44.82 | 13.15 | 8.80 | 33.23 | 78.05 | 0.1485 | -0.1981 |
|  | *Phlogotettix sp.* | 15136 | 42.73 | 12.80 | 9.34 | 35.13 | 77.86 | 0.0975 | -0.1561 |
|  | *Yanocephalus yanonis* | 15623 | 41.69 | 14.78 | 10.64 | 32.89 | 74.58 | 0.1180 | -0.1626 |
|  | *Scaphoideus maai* | 15188 | 41.84 | 13.16 | 9.59 | 35.41 | 77.25 | 0.0832 | -0.1568 |
|  | *Scaphoideusi nigrivalveus* | 15235 | 41.84 | 13.47 | 9.94 | 34.74 | 76.59 | 0.0927 | -0.1505 |
|  | *Scaphoideus varius* | 15207 | 40.81 | 14.19 | 9.94 | 35.06 | 75.87 | 0.0758 | -0.1760 |
|  | *Tambocerus sp.* | 15955 | 41.39 | 14.00 | 9.60 | 35.02 | 76.41 | 0.0834 | -0.1865 |
|  | *Maiestas dorsalis* | 15352 | 44.37 | 12.34 | 8.95 | 34.34 | 78.71 | 0.1274 | -0.1594 |
|  | *Japananus hyalinus* | 15364 | 42.66 | 13.69 | 9.73 | 33.92 | 76.58 | 0.1142 | -0.1692 |
|  | *Drabescoides nuchalis* | 15309 | 41.55 | 14.32 | 10.06 | 34.07 | 75.62 | 0.0989 | -0.1747 |
|  | *Macrosteles quadrimaculatus* | 15734 | 43.51 | 12.74 | 9.55 | 34.19 | 77.70 | 0.1199 | -0.1431 |
|  | *Macrosteles quadrilineatus* | 16626 | 43.60 | 12.57 | 9.39 | 34.43 | 78.03 | 0.1175 | -0.1446 |
|  | *Nephotettix cincticeps* | 14805 | 41.59 | 12.22 | 10.13 | 36.06 | 77.65 | 0.0712 | -0.0934 |
|  | *Paralaevicephalus gracilipenis* | 16114 | 42.04 | 14.31 | 10.27 | 33.38 | 75.42 | 0.1148 | -0.1641 |
|  | *Watanabella graminea* | 15011 | 42.39 | 13.33 | 10.36 | 33.92 | 76.31 | 0.1110 | -0.1254 |
| Idiocerinae | *Populicerus populi* | 16494 | 41.45 | 11.91 | 10.90 | 35.73 | 77.19 | 0.0741 | -0.0444 |
| Eurymelinae | *Idioscopus myrica* | 15423 | 43.04 | 12.44 | 9.69 | 34.83 | 77.87 | 0.1054 | -0.1245 |
|  | *Parocerus laurifoliae* | 16811 | 43.32 | 11.20 | 9.30 | 36.19 | 79.51 | 0.0896 | -0.0926 |
|  | *Idioscopus clypealis* | 15393 | 42.48 | 11.91 | 9.76 | 35.84 | 78.32 | 0.0848 | -0.0992 |
|  | *Idioscopus nitidulus* | 15287 | 43.60 | 12.10 | 9.25 | 35.06 | 78.65 | 0.1085 | -0.1333 |
| Iassinae | *Batracomorphus lateprocessus* | 15356 | 46.39 | 11.36 | 8.20 | 34.06 | 80.44 | 0.1532 | -0.1615 |
|  | *Krisna concava* | 14304 | 46.41 | 11.70 | 8.54 | 33.35 | 79.76 | 0.1636 | -0.1558 |
|  | *Krisna rufimarginata* | 14724 | 47.17 | 10.92 | 8.02 | 33.89 | 81.06 | 0.1638 | -0.1531 |
|  | *Gessius rufidorsus* | 14634 | 45.38 | 11.06 | 8.22 | 35.34 | 80.71 | 0.1244 | -0.1474 |
|  | *Trocnadella arisana* | 15131 | 45.91 | 11.27 | 8.02 | 34.80 | 80.70 | 0.1377 | -0.1685 |
|  | *Iassus dorsalis* | 15176 | 46.92 | 10.91 | 9.01 | 33.15 | 80.07 | 0.1720 | -0.0952 |
| Cicadellinae | *Bothrogonia ferruginea* | 15262 | 44.75 | 13.53 | 9.99 | 31.73 | 76.48 | 0.1702 | -0.1504 |
|  | *Homalodisca vitripennis* | 15304 | 43.00 | 12.09 | 9.53 | 35.38 | 78.38 | 0.0971 | -0.1182 |
|  | *Cicadella viridis* | 15891 | 41.33 | 11.78 | 10.13 | 36.76 | 78.09 | 0.0585 | -0.0756 |
| Coelidiinae | *Taharana fasciana* | 15161 | 45.16 | 13.39 | 8.74 | 32.71 | 77.87 | 0.1599 | -0.2101 |
|  | *Olidiana ritcheriina* | 15166 | 44.63 | 13.48 | 8.52 | 33.37 | 78.00 | 0.1443 | -0.2257 |
| Megophthalminae | *Japanagallia spinosa* | 15655 | 43.90 | 14.20 | 9.10 | 32.70 | 76.60 | 0.1462 | -0.2189 |
|  | *Durgades nigropicta* | 15974 | 44.89 | 12.28 | 8.93 | 33.91 | 78.79 | 0.1394 | -0.1576 |
| Mileewinae | *Mileewa albovittata* | 15079 | 43.67 | 12.01 | 8.38 | 35.95 | 79.61 | 0.0970 | -0.1783 |
| Macropsinae | *Macropsis notata* | 16323 | 44.34 | 13.15 | 10.00 | 32.51 | 76.84 | 0.1540 | -0.1360 |
|  | *Oncopsis nigrofasciata* | 15927 | 44.41 | 11.36 | 9.67 | 34.56 | 78.97 | 0.1248 | -0.0806 |
| Ledrinae | *Ledra auditura* | 16094 | 29.87 | 10.46 | 13.28 | 46.38 | 76.26 | **-0.2165** | **0.1186** |
|  | *Tituria pyramidata* | 15331 | 29.01 | 10.63 | 13.74 | 46.62 | 75.63 | **-0.2328** | **0.1274** |
| Evacanthinae | [*Evacanthus acuminatus*](https://www.ncbi.nlm.nih.gov/nuccore/MK948205.1) | 14793 | 40.04 | 11.16 | 10.03 | 38.77 | 78.81 | 0.0161 | -0.0533 |
|  | [*Evacanthus heimianus*](https://www.ncbi.nlm.nih.gov/nuccore/MG813486.1) | 15806 | 40.73 | 10.69 | 9.43 | 39.16 | 79.89 | 0.0197 | -0.0626 |
