# Supplementary material for: Structural features and phylogenetic implications of Cicadellidae subfamily and two new mitogenomes leafhoppers
Source: PLoS One. 2021 May 14;16(5):e0251207. doi: 10.1371/journal.pone.0251207 (PMC8121325; doi:10.1371/journal.pone.0251207)
Supplement: S2 Table — (DOCX) [file pone.0251207.s003.docx]

**S2 Table. Nucleotide composition, AT and GC skews calculatesd for the 37 mitochondrial genome of *E. wengangensis* and *E. gracilis*.**

| ***E. wengangensis*/*E. gracilis*** | | | | | | |
| --- | --- | --- | --- | --- | --- | --- |
| **Gene** | **A+T%** | | **AT-skew** | | **GC-skew** | |
| tRNA-*Ile* | 71.4 | 76.6 | 0.022 | 0.020 | 0.333 | 0.751 |
| tRNA-*Gln* | 71.0 | 77.1 | 0.020 | 0.037 | -0.300 | 0.321 |
| tRNA-*Met* | 68.6 | 73.2 | 0.042 | 0.038 | -0.182 | 0.211 |
| *nad2* | 78.3 | 80.4 | -0.030 | -0.110 | -0.223 | 0.461 |
| tRNA-*Trp* | 81.5 | 80.3 | 0.019 | 0.057 | -0.167 | 0.338 |
| tRNA-*Cys* | 83.9 | 90.8 | -0.038 | -0.051 | -0.400 | 0.398 |
| tRNA-*Tyr* | 73.1 | 77.8 | 0.143 | 0.020 | -0.222 | 0.167 |
| *cox1* | 70.4 | 69.1 | -0.049 | -0.089 | -0.037 | 0.481 |
| tRNA-*Leu* | 72.7 | 76.8 | 0.083 | 0.057 | 0.111 | 0.520 |
| *cox2* | 73.2 | 74.1 | 0.074 | 0.070 | -0.126 | 0.283 |
| tRNA-*Lys* | 69.0 | 70.4 | 0.020 | 0.000 | -0.091 | 0.442 |
| tRNA-*Asp* | 84.4 | 87.5 | 0.111 | 0.143 | 0.000 | 0.333 |
| *atp8* | 83.0 | 83.0 | 0.102 | 0.134 | -0.308 | 0.218 |
| *atp6* | 75.6 | 74.2 | -0.037 | 0.023 | -0.208 | 0.245 |
| *cox3* | 72.7 | 71.2 | -0.019 | -0.052 | -0.042 | 0.451 |
| tRNA-*Gly* | 82.3 | 79.0 | 0.098 | 0.143 | -0.091 | 0.442 |
| *nad3* | 77.4 | 80.5 | -0.029 | 0.025 | -0.300 | 0.063 |
| tRNA-*Ala* | 69.8 | 71.6 | -0.136 | -0.250 | 0.263 | 0.673 |
| tRNA-*Arg* | 77.3 | 77.8 | -0.020 | 0.061 | -0.067 | 0.290 |
| tRNA-*Asn* | 80.0 | 82.1 | 0.192 | 0.127 | 0.231 | 0.503 |
| tRNA-*Ser* | 70.0 | 63.2 | 0.048 | -0.070 | 0.222 | 0.573 |
| tRNA-*Glu* | 86.6 | 89.4 | 0.138 | 0.017 | -0.333 | 0.245 |
| tRNA-*Phe* | 81.8 | 80.6 | 0.000 | 0.074 | -0.667 | 0.013 |
| *nad5* | 78.1 | 78.1 | 0.340 | 0.320 | -0.235 | 0.137 |
| tRNA-*His* | 82.5 | 81.2 | 0.077 | 0.179 | -0.636 | -0.229 |
| *nad4* | 78.4 | 76.9 | 0.335 | 0.373 | -0.154 | 0.134 |
| *nad4L* | 82.1 | 79.6 | 0.345 | 0.369 | -0.360 | -0.043 |
| tRNA-*Thr* | 85.9 | 83.9 | 0.018 | 0.038 | 0.111 | 0.210 |
| tRNA-*Pro* | 78.1 | 76.5 | 0.200 | 0.192 | -0.429 | -0.155 |
| *nad6* | 82.0 | 80.9 | -0.010 | 0.140 | -0.195 | 0.137 |
| *cytb* | 70.1 | 72.6 | -0.059 | 0.039 | -0.141 | 0.293 |
| tRNA-*Ser* | 81.0 | 82.1 | 0.020 | 0.091 | 0.333 | 0.740 |
| *nad1* | 74.2 | 77.9 | 0.330 | 0.396 | -0.128 | 0.011 |
| tRNA-*Leu* | 73.8 | 79.1 | 0.083 | 0.170 | -0.412 | 0.091 |
| *16S* | 81.9 | 82.9 | 0.159 | 0.235 | -0.237 | 0.024 |
| tRNA-*Val* | 81.3 | 76.9 | 0.115 | 0.120 | 0.000 | 0.453 |
| *12S* | 79.8 | 80.3 | 0.155 | 0.244 | -0.265 | 0.144 |
