# Supplementary material for: Structural features and phylogenetic implications of Cicadellidae subfamily and two new mitogenomes leafhoppers
Source: PLoS One. 2021 May 14;16(5):e0251207. doi: 10.1371/journal.pone.0251207 (PMC8121325; doi:10.1371/journal.pone.0251207)
Supplement: S3 Table — (DOCX) [file pone.0251207.s004.docx]

**S3 Table. 13 PCGs nucleotide compositions, AT- skews and GC-skews in 56 species of Cicadellidae.**

| **Subfamily** | **Species** | **Length(bp)** | **A** | **C** | **G** | **T(U)** | **A+T%** | **AT-skew** | **GC-skew** | **Pos1%** | **Pos2%** | **Pos3%** |
| --- | --- | --- | --- | --- | --- | --- | --- | --- | --- | --- | --- | --- |
| Typhlocybinae | *Empoascanara wengangensis* | 10964 | 42.19 | 14.16 | 10.46 | 33.19 | 75.38 | 0.1194 | -0.1501 | 71.93 | 73.38 | 80.84 |
|  | *Empoascanara gracilis* | 10976 | 42.90 | 14.09 | 10.27 | 32.74 | 75.64 | 0.1344 | -0.1571 | 71.58 | 74.42 | 80.92 |
|  | *Empoascanara dwalata* | 10955 | 41.12 | 13.73 | 11.16 | 33.98 | 75.11 | 0.0950 | -0.1030 | 71.58 | 73.36 | 80.39 |
|  | *Empoascanara sipra* | 10938 | 42.08 | 14.09 | 10.54 | 33.29 | 75.37 | 0.1167 | -0.1440 | 77.37 | 70.32 | 78.41 |
|  | *Mitjaevia protuberanta* | 10969 | 38.40 | 12.75 | 11.73 | 37.11 | 75.51 | 0.0170 | -0.0417 | 76.92 | 76.23 | 73.39 |
|  | *Limassolla lingchuanensis* | 10943 | 42.50 | 12.90 | 10.80 | 33.70 | 76.20 | 0.1155 | -0.0886 | 74.80 | 75.30 | 78.60 |
|  | *Paraahimia luodianensis* | 10920 | 45.63 | 13.04 | 9.26 | 32.07 | 77.70 | 0.1745 | -0.1696 | 81.21 | 70.77 | 81.13 |
|  | *Zyginella minuta* | 10928 | 41.80 | 15.03 | 11.15 | 32.01 | 73.81 | 0.1327 | -0.1481 | 69.81 | 72.80 | 78.83 |
|  | *Parathailocyba orla* | 10929 | 45.57 | 13.61 | 9.53 | 31.30 | 76.87 | 0.1856 | -0.1764 | 80.48 | 70.13 | 79.99 |
|  | *Typhlocyba sp.* | 10952 | 43.19 | 14.14 | 10.70 | 31.97 | 75.16 | 0.1493 | -0.1386 | 72.67 | 73.40 | 79.40 |
|  | *Eupteryx minuscula* | 10924 | 42.59 | 12.80 | 11.10 | 33.51 | 76.10 | 0.1192 | -0.0709 | 77.13 | 75.75 | 75.42 |
|  | *Bolanusoides shaanxiensis* | 10921 | 45.16 | 13.01 | 9.78 | 32.05 | 77.21 | 0.1698 | -0.1418 | 80.01 | 74.70 | 76.92 |
|  | *Empoasca vitis* | 10947 | 36.17 | 11.67 | 11.56 | 40.61 | 76.78 | -0.0579 | -0.0047 | 77.61 | 73.50 | 79.23 |
|  | *Ghauriana sinensis* | 10930 | 36.70 | 10.00 | 10.60 | 42.70 | 79.40 | -0.0756 | 0.0291 | 79.00 | 82.40 | 76.60 |
|  | *Empoasca flavescens* | 10947 | 36.17 | 11.67 | 11.56 | 40.61 | 76.78 | -0.0579 | -0.0047 | 77.61 | 73.50 | 79.23 |
|  | *Empoasca onukii* | 10701 | 36.04 | 11.77 | 11.64 | 40.54 | 76.58 | -0.0587 | -0.0056 | 78.27 | 73.45 | 78.02 |
| Deltocephalinae | *Pellucidus guizhouensis sp.* | 10980 | 44.63 | 14.85 | 9.62 | 30.91 | 75.54 | 0.1816 | -0.2137 | 76.61 | 71.37 | 78.63 |
|  | *Phlogotettix sp.* | 10952 | 42.45 | 13.33 | 9.75 | 34.47 | 76.92 | 0.1038 | -0.1551 | 68.15 | 77.95 | 84.66 |
|  | *Yanocephalus yanonis* | 10937 | 41.32 | 15.69 | 11.24 | 31.75 | 73.07 | 0.1309 | -0.1654 | 71.48 | 73.09 | 74.65 |
|  | *Scaphoideus maai* | 10929 | 41.63 | 13.73 | 9.91 | 34.72 | 76.36 | 0.0905 | -0.1618 | 76.50 | 70.38 | 82.19 |
|  | *Scaphoideusi nigrivalveus* | 10934 | 41.64 | 14.10 | 10.20 | 34.06 | 75.70 | 0.1002 | -0.1607 | 73.20 | 75.23 | 78.68 |
|  | *Scaphoideus varius* | 10938 | 40.34 | 14.90 | 10.39 | 34.38 | 74.71 | 0.0798 | -0.1786 | 73.83 | 74.22 | 76.08 |
|  | *Tambocerus sp.* | 11155 | 39.30 | 15.42 | 10.69 | 34.59 | 73.89 | 0.0638 | -0.1809 | 72.79 | 73.70 | 75.17 |
|  | *Maiestas dorsalis* | 10961 | 44.27 | 12.72 | 9.21 | 33.80 | 78.07 | 0.1340 | -0.1597 | 79.53 | 76.85 | 77.83 |
|  | *Japananus hyalinus* | 10953 | 42.70 | 14.33 | 9.85 | 33.11 | 75.81 | 0.1264 | -0.1854 | 78.58 | 69.02 | 79.84 |
|  | *Drabescoides nuchalis* | 10933 | 41.16 | 14.88 | 10.50 | 33.46 | 74.62 | 0.1032 | -0.1726 | 74.27 | 71.68 | 77.91 |
|  | *Macrosteles quadrimaculatus* | 10966 | 42.84 | 13.77 | 10.32 | 33.07 | 75.91 | 0.1288 | -0.1431 | 73.93 | 75.43 | 78.36 |
|  | *Macrosteles quadrilineatus* | 10942 | 43.03 | 13.68 | 9.97 | 33.32 | 76.35 | 0.1271 | -0.1569 | 74.48 | 76.28 | 78.28 |
|  | *Nephotettix cincticeps* | 10901 | 40.92 | 12.51 | 10.64 | 35.92 | 76.85 | 0.0651 | -0.0808 | 69.68 | 74.05 | 86.82 |
|  | *Paralaevicephalus gracilipenis* | 10941 | 41.54 | 15.43 | 10.78 | 32.25 | 73.80 | 0.1258 | -0.1775 | 74.99 | 69.18 | 77.21 |
|  | *Watanabella graminea* | 10946 | 41.99 | 13.84 | 10.56 | 33.61 | 75.60 | 0.1108 | -0.1344 | 74.05 | 74.62 | 78.13 |
| Idiocerinae | *Populicerus populi* | 10941 | 32.61 | 12.21 | 12.14 | 43.04 | 75.65 | -0.1379 | -0.0030 | 76.34 | 75.79 | 74.83 |
| Eurymelinae | *Idioscopus myrica* | 10936 | 32.49 | 11.22 | 12.14 | 44.15 | 76.64 | -0.1521 | 0.0395 | 75.86 | 73.61 | 80.44 |
|  | *Parocerus laurifoliae* | 10945 | 33.23 | 10.85 | 11.25 | 44.67 | 77.90 | -0.1468 | 0.0178 | 77.39 | 74.62 | 81.69 |
|  | *Idioscopus clypealis* | 10945 | 32.53 | 10.80 | 11.81 | 44.86 | 77.39 | -0.1594 | 0.0448 | 78.98 | 74.12 | 79.06 |
|  | *Idioscopus nitidulus* | 10943 | 42.69 | 12.76 | 9.88 | 34.67 | 77.36 | 0.1037 | -0.1272 | 71.88 | 79.85 | 80.37 |
| Iassinae | *Batracomorphus lateprocessus* | 10901 | 46.16 | 11.82 | 8.58 | 33.44 | 79.60 | 0.1598 | -0.1592 | 75.51 | 81.37 | 81.92 |
|  | *Krisna concava* | 10955 | 45.52 | 12.20 | 9.01 | 33.26 | 78.79 | 0.1556 | -0.1506 | 74.89 | 79.82 | 81.65 |
|  | *Krisna rufimarginata* | 10946 | 46.88 | 11.52 | 8.30 | 33.31 | 80.18 | 0.1692 | -0.1627 | 75.47 | 82.10 | 82.98 |
|  | *Gessius rufidorsus* | 10938 | 44.95 | 11.51 | 8.49 | 35.04 | 80.00 | 0.1239 | -0.1508 | 81.38 | 79.46 | 79.16 |
|  | *Trocnadella arisana* | 10902 | 46.45 | 11.76 | 8.55 | 33.24 | 79.69 | 0.1657 | -0.1581 | 81.73 | 74.71 | 82.64 |
|  | *Iassus dorsalis* | 10898 | 46.75 | 11.71 | 9.13 | 32.41 | 79.16 | 0.1812 | -0.1237 | 73.22 | 79.82 | 84.44 |
| Cicadellinae | *Bothrogonia ferruginea* | 10920 | 32.91 | 12.78 | 12.24 | 42.06 | 74.97 | -0.1220 | -0.0216 | 75.14 | 73.87 | 75.91 |
|  | *Homalodisca vitripennis* | 10963 | 33.06 | 11.11 | 11.72 | 44.11 | 77.17 | -0.1433 | 0.0268 | 81.83 | 74.88 | 74.79 |
|  | *Cicadella viridis* | 10923 | 40.72 | 12.41 | 10.64 | 36.24 | 76.96 | 0.0583 | -0.0767 | 79.92 | 70.48 | 80.47 |
| Coelidiinae | *Taharana fasciana* | 10874 | 44.87 | 14.36 | 9.12 | 31.65 | 76.52 | 0.1727 | -0.2229 | 81.63 | 73.52 | 74.42 |
|  | *Olidiana ritcheriina* | 10898 | 44.70 | 14.41 | 8.74 | 32.15 | 76.85 | 0.1632 | -0.2446 | 73.49 | 75.09 | 81.97 |
| Megophthalminae | *Japanagallia spinosa* | 10915 | 43.79 | 15.36 | 9.88 | 30.97 | 74.76 | 0.1716 | -0.2174 | 76.26 | 74.82 | 73.20 |
|  | *Durgades nigropicta* | 10934 | 44.86 | 12.98 | 9.26 | 32.90 | 77.76 | 0.1538 | -0.1669 | 78.44 | 77.50 | 77.33 |
| Mileewinae | *Mileewa albovittata* | 10961 | 43.50 | 12.70 | 8.74 | 35.06 | 78.56 | 0.1074 | -0.1847 | 71.15 | 79.17 | 85.35 |
| Macropsinae | *Macropsis notata* | 10991 | 43.64 | 14.74 | 10.86 | 30.75 | 74.40 | 0.1733 | -0.1514 | 74.32 | 72.71 | 76.17 |
|  | *Oncopsis nigrofasciata* | 10953 | 44.10 | 12.12 | 10.42 | 33.37 | 77.47 | 0.1385 | -0.0754 | 79.92 | 68.99 | 83.48 |
| Ledrinae | *Ledra auditura* | 10952 | 27.33 | 10.95 | 14.47 | 47.25 | 74.58 | -0.2671 | 0.1386 | 71.27 | 73.49 | 78.99 |
|  | *Tituria pyramidata* | 10920 | 26.62 | 10.61 | 15.25 | 47.52 | 74.14 | -0.2819 | 0.1792 | 75.80 | 68.93 | 77.69 |
| Evacanthinae | [*Evacanthus acuminatus*](https://www.ncbi.nlm.nih.gov/nuccore/MK948205.1) | 10944 | 38.73 | 11.15 | 10.36 | 39.76 | 78.49 | -0.0130 | -0.0365 | 79.52 | 73.60 | 82.35 |
|  | [*Evacanthus heimianus*](https://www.ncbi.nlm.nih.gov/nuccore/MG813486.1) | 10950 | 39.10 | 10.97 | 10.05 | 39.89 | 78.99 | -0.0101 | -0.0439 | 80.33 | 73.81 | 82.82 |
