# Supplementary material for: Structural features and phylogenetic implications of Cicadellidae subfamily and two new mitogenomes leafhoppers
Source: PLoS One. 2021 May 14;16(5):e0251207. doi: 10.1371/journal.pone.0251207 (PMC8121325; doi:10.1371/journal.pone.0251207)
Supplement: S4 Table — (DOCX) [file pone.0251207.s005.docx]

**S4 Table. Codon and relative synonymous codon usage (RSCU) of 13 PCGs in the mt genomes of *E. wengangensis* and *E. gracilis.***

| **Amino**  **Acid** | ***Codon*** | ***Count/RSCU*** | | | | **Amino**  **Acid** | ***Codon*** | ***Count/RSCU*** | | | |
| --- | --- | --- | --- | --- | --- | --- | --- | --- | --- | --- | --- |
|  |  | ***E. wengangesis*** | | ***E. gracilis*** | |  |  | ***E. wengangesis*** | | ***E. gracilis*** | |
| Phe | UUU | **161** | **1.5** | **141** | **1.5** | Tyr | UAU | **117** | **1.45** | **111** | **1.42** |
|  | UUC | 53 | 0.5 | 47 | 0.5 |  | UAC | 44 | 0.55 | 45 | 0.58 |
| Leu2 | UUA | **206^a^** | **3.07** | **196** | **2.94** | His | CAU | **53** | **1.41** | **63** | **1.4** |
|  | UUG | 32 | 0.48 | 39 | 0.58 |  | CAC | 22 | 0.59 | 27 | 0.6 |
| Leu1 | CUU | **54** | **0.8** | **68** | **1.02** | Gln | CAA | **90** | **1.64** | **110** | **1.71** |
|  | CUC | 12 | 0.18 | 16 | 0.24 |  | CAG | 20 | 0.36 | 19 | 0.29 |
|  | CUA | 79 | 1.18 | 66 | 0.99 | Asn | AAU | **240** | **1.54** | **261** | **1.57** |
|  | CUG | 20 | 0.3 | 15 | 0.23 |  | AAC | 72 | 0.46 | 71 | 0.43 |
| Ile | AUU | **257** | **1.62** | **263** | **1.67** | Lys | AAA | **329** | **1.8** | **324** | **1.77** |
|  | AUC | 61 | 0.38 | 52 | 0.33 |  | AAG | 36 | 0.2 | 43 | 0.23 |
| Met | AUA | **211** | **1.7** | **206** | **1.73** | Asp | GAU | **44** | **1.47** | **56** | **1.65** |
|  | AUG | 37 | 0.3 | 32 | 0.27 |  | GAC | 16 | 0.53 | 12 | 0.35 |
| Val | GUU | 35 | 1.33 | 33 | 1.22 | Glu | GAA | **111** | **1.66** | **111** | **1.72** |
|  | GUC | 9 | 0.34 | 11 | 0.41 |  | GAG | 23 | 0.34 | 18 | 0.28 |
|  | GUA | **53** | **2.02** | **50** | **1.85** | Cys | UGU | **24** | **1.33** | **18** | **1.29** |
|  | GUG | 8 | 0.3 | 14 | 0.52 |  | UGC | 12 | 0.67 | 10 | 0.71 |
| Ser2 | UCU | 54 | 1.5 | 42 | 1.21 | Trp | UGA | **65** | **1.71** | **67** | **1.65** |
|  | UCC | 14 | 0.39 | 23 | 0.66 |  | UGG | 11 | 0.29 | 14 | 0.35 |
|  | UCA | **102** | **2.83** | **95** | **2.73** | Arg | CGU | 13 | 1.06 | 9 | 0.84 |
|  | UCG | 6 | 0.17 | 8 | 0.23 |  | CGC | 1 | 0.08 | 3 | 0.28 |
| Pro | CCU | **61** | **1.73** | **48** | **1.41** |  | CGA | **31** | **2.53** | **27** | **2.51** |
|  | CCC | 21 | 0.6 | 24 | 0.71 |  | CGG | 4 | 0.33 | 4 | 0.37 |
|  | CCA | 53 | 1.5 | 57 | 1.68 | Ser1 | AGU | 29 | 0.81 | 28 | 0.81 |
|  | CCG | 6 | 0.17 | 7 | 0.21 |  | AGC | 18 | 0.5 | 20 | 0.58 |
| Thr | ACU | **76** | **1.55** | **79** | **1.6** |  | AGA | **47** | **1.31** | **42** | **1.21** |
|  | ACC | 43 | 0.88 | 31 | 0.63 |  | AGG | 18 | 0.5 | 20 | 0.58 |
|  | ACA | 68 | 1.39 | 79 | 1.6 | Gly | GGU | 25 | 0.98 | 39 | 1.51 |
|  | ACG | 9 | 0.18 | 9 | 0.18 |  | GGC | 4 | 0.16 | 10 | 0.39 |
| Ala | GCU | 30 | 1.41 | 26 | 1.51 |  | GGA | **46** | **1.8** | **38** | **1.48** |
|  | GCC | 12 | 0.56 | 11 | 0.64 |  | GGG | 27 | 1.06 | 16 | 0.62 |
|  | GCA | **38** | **1.79** | **28** | **1.62** | * | UAA | 152 | 1.73 | 179 | 1.77 |
|  | GCG | 5 | 0.24 | 4 | 0.23 |  | UAG | 24 | 0.27 | 23 | 0.23 |

a The higher values of preferentially used codons are in bold.
